# The Influence of Phosphorus Substituents on the Structures and Solution Speciation of Trivalent Uranium and Lanthanide Phosphinodiboranates

**DOI:** 10.1021/acs.inorgchem.3c02773

**Published:** 2023-11-27

**Authors:** Joshua C. Zgrabik, Rina Bhowmick, Francesca D. Eckstrom, A. Rayford Harrison, Taylor V. Fetrow, Anastasia V. Blake, Bess Vlaisavljevich, Scott R. Daly

**Affiliations:** † Department of Chemistry, The University of Iowa, E331 Chemistry Building, Iowa City, Iowa 52242, United States; ‡ Department of Chemistry, The University of South Dakota, 414 E Clark St., Vermillion, South Dakota 57069, United States

## Abstract

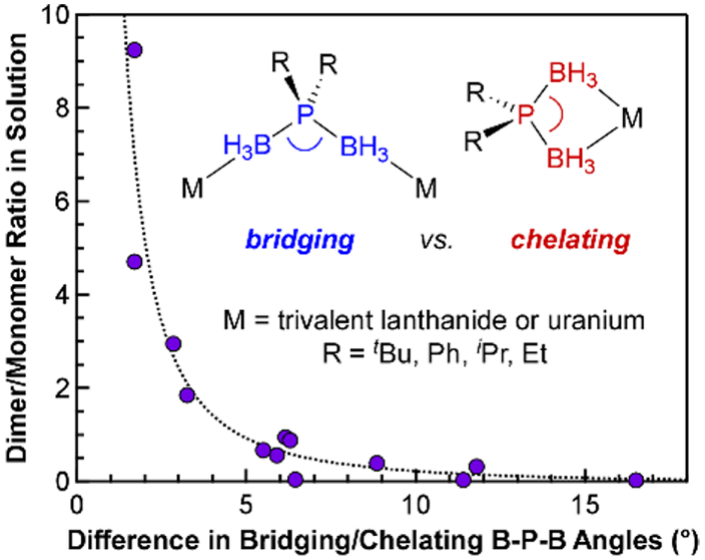

Here, we report the mechanochemical synthesis and characterization of homoleptic uranium and lanthanide phosphinodiboranates with isopropyl and ethyl substituents attached to phosphorus. M(H_3_BP^*i*^Pr_2_BH_3_)_3_ complexes with M = U, Nd, Sm, Tb, and Er were prepared by ball milling UI_3_(THF)_4_, SmBr_3_, or MI_3_ with three equivalents of K(H_3_BP^*i*^Pr_2_BH_3_). M(H_3_BPEt_2_BH_3_)_3_ with M = U and Nd were prepared similarly using K(H_3_BPEt_2_BH_3_), and the complexes were purified by extraction and crystallization from Et_2_O or CH_2_Cl_2_. Single-crystal XRD studies revealed that all five M(H_3_BP^*i*^Pr_2_BH_3_)_3_ crystallize as dimers, despite the significant differences in metal radii across the series. In contrast, Nd(H_3_BPEt_2_BH_3_)_3_ with smaller ethyl substituents crystallized as a coordination polymer. Crystals of U(H_3_BPEt_2_BH_3_)_3_ were not suitable for structural analysis, but crystals of U(H_3_BPMe_2_BH_3_)_3_ isolated in low yield by solution methods were isostructural with Nd(H_3_BPEt_2_BH_3_)_3_. ^1^H and ^11^B NMR studies in C_6_D_6_ revealed that all of the complexes form mixtures of monomer and oligomers when dissolved, and the extent of oligomerization was highly dependent on metal radius and phosphorus substituent size. A comprehensive analysis of all structurally characterized uranium and lanthanide phosphinodiboranate complexes reported to date, including those with larger Ph and ^*t*^Bu substituents, revealed that the degree of oligomerization in solution can be correlated to differences in B–P–B angles obtained from single-crystal XRD studies. Density functional theory calculations, which included structural optimizations in combination with conformational searches using tight binding methods, replicated the general experimental trends and revealed free energy differences that account for the different solution and solid-state structures. Collectively, these results reveal how steric changes to phosphorus substituents significantly removed from metal coordination sites can have a significant influence on solution speciation, deoligomerization energies, and the solid-state structure of homoleptic phosphinodiboranate complexes containing trivalent f-metals.

## Abstract 2

Here, we report the mechanochemical synthesis and characterization of homoleptic uranium and lanthanide phosphinodiboranate with isopropyl and ethyl substituents attached to phosphorus. Comparison of the M(H_3_BPR_2_BH_3_)_3_ complexes (M = U or lanthanides) with R = ^*i*^Pr and Et to those reported previously with R = ^*t*^Bu and Ph reveals how steric changes significantly removed from the metal can have a significant influence on solution speciation, deoligomerization energies, and solid-state structure of phosphinodiboranate complexes.

## Introduction

**Scheme 1:**
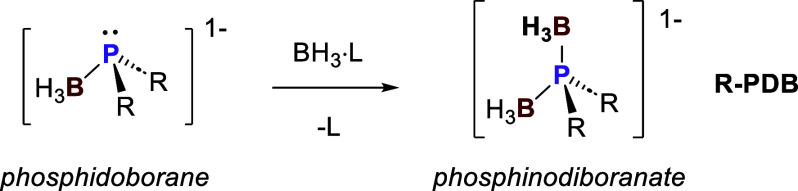
General Synthesis and Structure of Phosphinodiboranates (Which Are Also Referred to As Phosphido-Bis(borane) Anions)

Phosphinodiboranates are monoanionic borohydrides that have the general formula H_3_BPR_2_BH_3_^–^ (abbreviated here as R-PDB, where R is the substituent attached to phosphorus; Scheme 1).

**Scheme 2:**
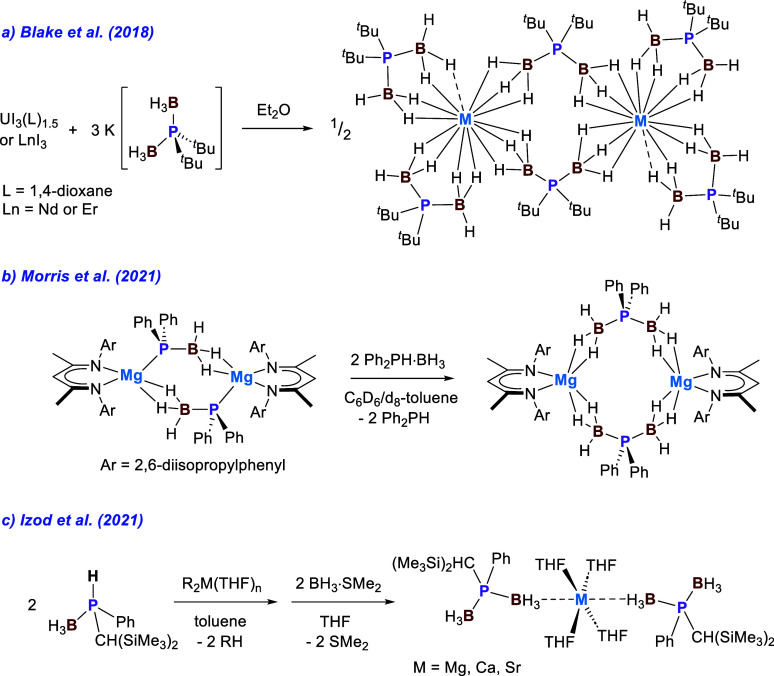
(a) Initial Salt Metathesis Reactions Used to Prepare M-^*t*^Bu with M = U, Nd, and Er;^14^ (b) Synthesis of [(BDI)Mg(H_3_BPPh_2_BH_3_)]_2_ via Borane Transfer to the Phosphidoborane Ligand in [(BDI)Mg(H_3_BPPh_2_)]_2_;^15^ (c) Synthesis of (H_3_BPR^1^R^2^BH_3_)_2_M(THF)_4_ Complexes with R^1^ = Ph; R^2^ = CH(SiMe_3_)_2_; and M = Mg, Ca, and Sr^17^

Despite being known to form salts with alkali metals with different substituents attached to phosphorus since at least the 1960s,^1−11^ and likely as early as 1940,^12^ the coordinative properties of phosphinodiboranates with different metals have only emerged recently.^13^ In 2018, we showed that M(H_3_BP^*t*^Bu_2_BH_3_)_3_ (M-^*t*^Bu), where M = uranium or a lanthanide, can be prepared by salt metathesis reactions using trivalent f-metal iodides and K(H_3_BP^*t*^Bu_2_BH_3_) (Scheme 2a).^14^ Later, in 2021, Morris et al. reported the first magnesium complex containing Ph-PDB.^15^ The β-diketoiminate-supported complex [(BDI)Mg(H_3_BPPh_2_BH_3_)]_2_ where BDI = HC[C(CH_3_)Ndipp]_2_ and dipp = 2,6-^*i*^Pr_2_C_6_H_3_ was prepared by treating the phosphidoborane complex [(BDI)Mg(H_3_BPPh_2_)]_2_ with 2 equiv of HPPh_2_·BH_3_ (Scheme 2b).^15^ Incidentally, similar borane transfer reactivity was implicated in the formation of U(H_3_BP^*t*^Bu_2_BH_3_)_3_ from reactions of UI_3_(1,4-dioxane)_1.5_ and K(H_3_BP^*t*^Bu_2_), where H_3_BP^*t*^Bu_2_BH_3_^–^ is presumably formed via borane transfer from another unit of H_3_BP^*t*^Bu_2_^–^.^16^ Shortly after the report by Morris et al., Izod et al. reported how (H_3_BPR^1^R^2^BH_3_)_2_M(THF)_4_ complexes with R^1^ = Ph; R^2^ = CH(SiMe_3_)_2_; and M = Mg, Ca, and Sr could be prepared in a similar stepwise route by treating dialkyl metal complexes like ^*n*^Bu_2_Mg with 2 equiv of R_1_R_2_PH·BH_3_ followed by 2 equiv of BH_3_·SMe_2_ (Scheme 2c).^17^

As noted by us with trivalent uranium and lanthanides,^14^ and by Izod et al. with alkaline earth metals,^17^ metathesis reactions between metal iodides and lithium or potassium PDB salts do not proceed cleanly and are often low yielding in solvents such as Et_2_O and THF. This likely accounts for why coordination complexes with phosphinodiboranate ligands have only emerged within the past decade. Recently, we reported how mechanochemical grinding^18,19^ could be used to overcome these issues and form M-^*t*^Bu complexes in higher and more reproducible yields via salt metathesis reactions.^16^ In addition to facilitating more routine access to pure ^*t*^Bu-PDB complexes, mechanochemical methods also allowed access to M(H_3_BPPh_2_BH_3_)_3_ (M-Ph) complexes for the first time for comparative structural and spectroscopic analysis.^20^

Almost all the M-^*t*^Bu and M-Ph complexes reported to date form dimeric structures in the solid state,^16,20,21^ which is unusual given the significant differences in ionic radii ranging from La^3+^ (1.032 Å) and U^3+^ (1.025 Å) to Lu^3+^ (0.861 Å).^22^ The only exceptions we have observed thus far are Ph-PDB complexes with U^3+^ and Ce^3+^; Ce-Ph is polymeric in the solid state, whereas both crystals of dimeric and polymeric U-Ph have been isolated.^20^ Despite their solid-state similarities, more significant structural differences are observed for PDB complexes in solution. ^1^H and ^11^B NMR studies have shown that dimeric PDB complexes break up into metal-dependent mixtures of monomers and dimers (or higher order oligomers) when dissolved in aromatic solvents. M-^*t*^Bu complexes with the largest metal ions such as U^3+^, Ce^3+^, and Nd^3+^ appear to exist primarily as dimers in solution while smaller metal ions like Tb^3+^, Er^3+^, and Lu^3+^ exist primarily as monomers.^21^ M-Ph complexes with U^3+^ and the larger lanthanide ions Ce^3+^, Pr^3+^, and Nd^3+^ also appear to exist primarily as dimers when dissolved. However, it was not clear from these studies how the size of the substituents attached to phosphorus affects the degree of oligomerization in solution.

To more rigorously investigate the influence that phosphorus substituents have on the structure and reactivity of f-element phosphinodiboranates, we expanded our initial investigation of M-^*t*^Bu and M-Ph complexes reported previously to those reported here for the first time with smaller phosphorus substituents (M-^*i*^Pr and M-Et). Our goal was to determine how a stepwise decrease in substituent size, especially from ^*t*^Bu to ^*i*^Pr to Et, affects the solid state and solution structures of f-element PDB complexes.^23^ Here, we report the synthesis and characterization of ^*i*^Pr-PDB complexes with U^3+^ (U-^*i*^Pr), Nd^3+^ (Nd-^*i*^Pr), Sm^3+^ (Sm-^*i*^Pr), Tb^3+^ (Tb-^*i*^Pr), and Er^3+^ (Er-^*i*^Pr) as well as Et-PDB complexes with U^3+^ (U-Et) and Nd^3+^ (Nd-Et). Our results show how steric and metal-size-induced variations in B–P–B angles, as quantified using single-crystal XRD and supporting density functional theory (DFT) calculations, appear to control the extent of oligomerization of trivalent f-element PDB complexes in solution.

## Results and Discussion

### Synthesis and XRD Structures

**Scheme 3:**
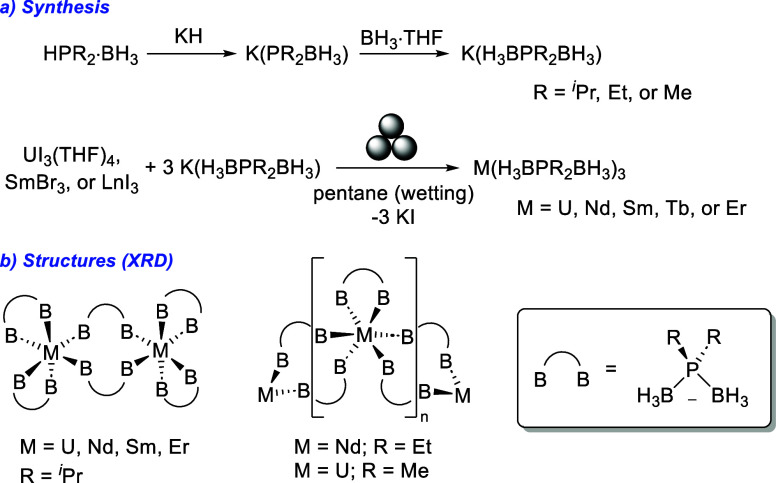
(a) General Synthesis of R-PDB Ligands and Metal Complexes and (b) Cartoon Showing the Arrangement of PDB Ligands and Degree of Solid-State Oligomerization Observed As a Function of Metal and Phosphorus Substituent Identity We note that B represents the relative location of the BH_3_ groups and does not indicate anything about their denticity.

The ligand starting materials K(H_3_BP^*i*^Pr_2_BH_3_) (^*i*^Pr-PDB) and K(H_3_BPEt_2_BH_3_) (Et-PDB) were synthesized by treating HP^*i*^Pr_2_·BH_3_ and HPEt_2_·BH_3_ with KH to form the phosphidoboranate, followed by the addition of BH_3_·THF (Scheme 3a).^6,7,24^ The M(H_3_BP^*i*^Pr_2_BH_3_)_3_ and M(H_3_BPEt_2_BH_3_)_3_ complexes (referred to as M-^*i*^Pr and M-Et, respectively, hereafter, where M = U or lanthanide) were prepared by ball milling 3 equiv of the corresponding potassium salt with UI_3_(THF)_4_, SmBr_3_, or LnI_3_ with several drops of pentane for wetting.^25^ After grinding, the products were extracted and crystallized from Et_2_O or CH_2_Cl_2_, typically by vapor diffusion with pentane, in low to moderate yields (14–57%). As discussed below, the complexes adopt different structures in solution and the solid state (Scheme 3b), so we have used empirical formulas throughout when referencing the complexes for consistency.

Single-crystal XRD studies revealed that M-^*i*^Pr complexes with M = U, Nd, Sm, Tb, and Er crystallize in the monoclinic *P*2_1_/*c* space group and form isostructural dimers, as observed previously for the M(H_3_BP^*t*^Bu_2_BH_3_)_3_ complexes (M-^*t*^Bu) with M = U^3+^ or Ln^3+^. However, there are distinct structural differences due to the changes in alkyl substituent. For reference, M-^*t*^Bu complexes maintain relatively symmetric bridging ^*t*^Bu-PDB ligands with similar M–B distances to both metals.^21^ They also alter the denticity of their chelating ligands from κ^2^-BH_3_ to κ^1^-BH_3_ to accommodate smaller metals.

**Figure 1:**
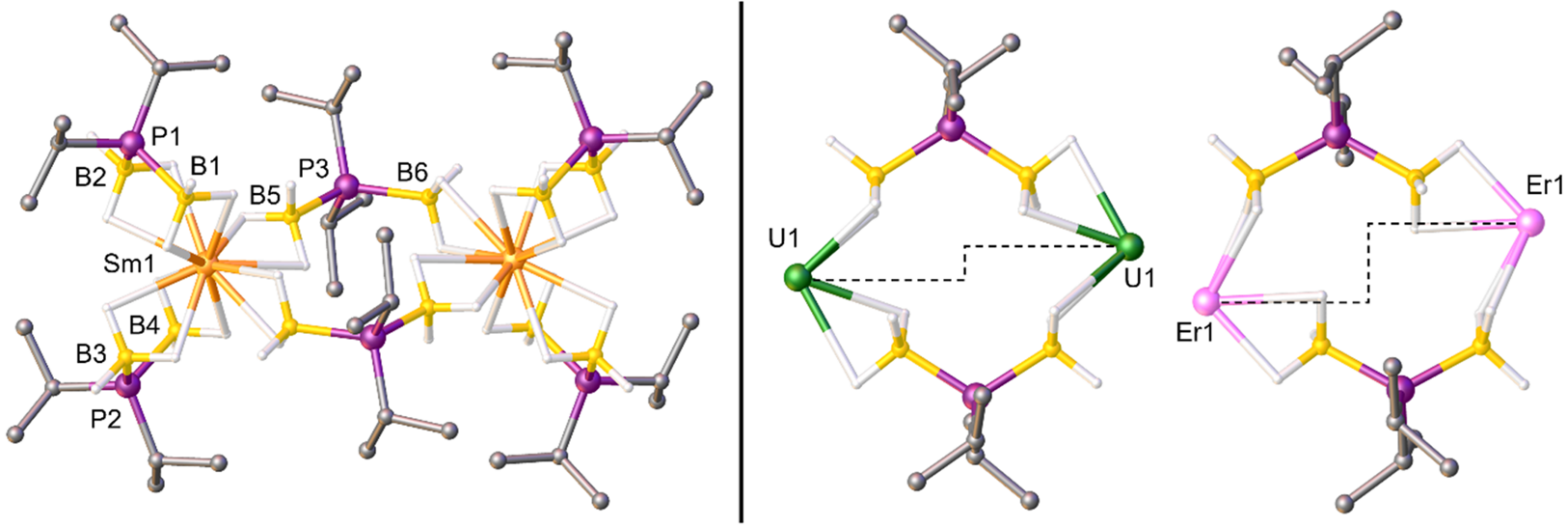
Left: Dimeric structure of Sm(H_3_BP^*i*^Pr_2_BH_3_)_3_ (Sm-^*i*^Pr) from single-crystal XRD studies. Right: Comparison of bridging PDB ligands in the dimeric XRD structures of U(H_3_BP^*i*^Pr_2_BH_3_)_3_ (U-^*i*^Pr) and Er(H_3_BP^*i*^Pr_2_BH_3_)_3_ (Er-^*i*^Pr). The dashed line helps to emphasize the increasing asymmetry in the bridging ^*i*^Pr-PDB ligand with respect to metal binding. Hydrogen atoms attached to carbon were omitted. Ball and stick representation shown for easier viewing of all the atoms.

**Figure 2:**
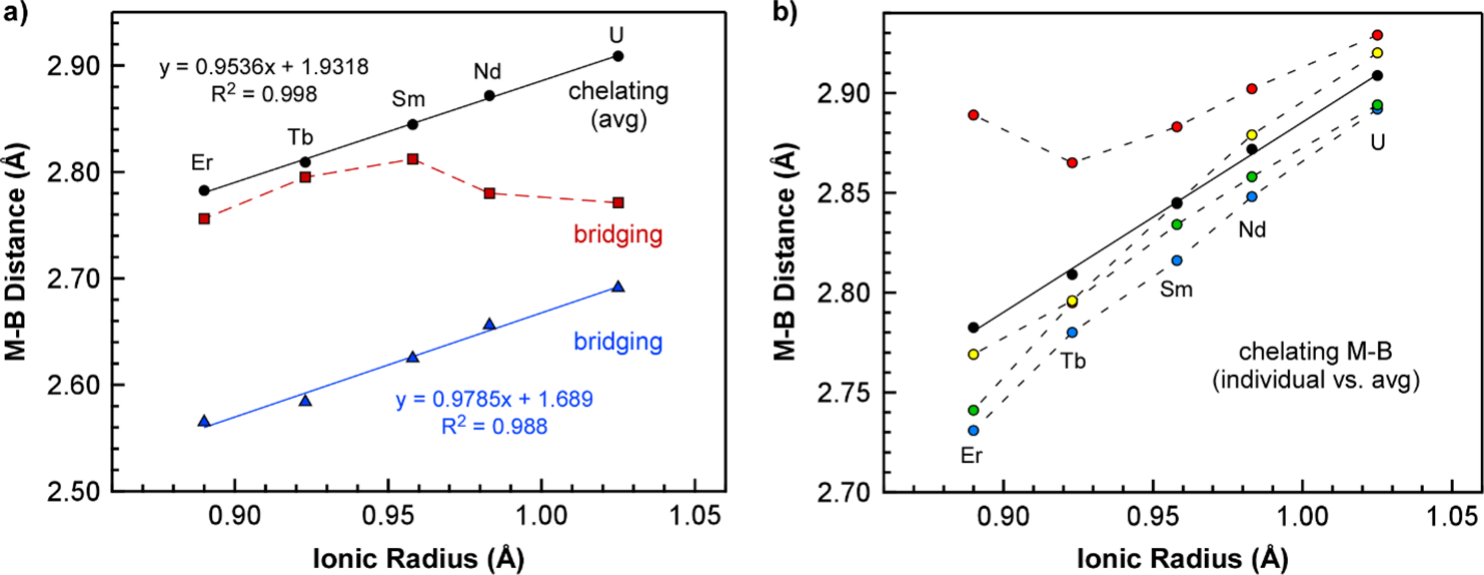
(a) Plot of chelating (average) and bridging M–B distances obtained from single-crystal XRD studies of M(H_3_BP^*i*^Pr_2_BH_3_)_3_ (M-^*i*^Pr) complexes vs ionic radius of the corresponding metal (CN = 6).^22^ The distances associated with the bridging ^*i*^Pr-PDB ligands are denoted by red squares and blue triangles, and average distances associated with the chelating ^*i*^Pr-PDB ligands are represented by black circles. (b) Plot of all chelating M–B distances (colored circles) and average chelating M–B distances (black circles) vs ionic radius of the metal. Solid lines in both plots represent linear fits, whereas dashed lines are included to help guide the eye between data points.

In contrast to M-^*t*^Bu complexes, M-^*i*^Pr complexes adjust the denticity of their bridging ligands (not chelating) to accommodate different size metals, and the bridging ligands are asymmetric with one short M–B distance and one significantly longer M–B distance (Figure 1). The short M–B distances are consistent with those of κ^3^-BH_3_, and they display a clear linear correlation when plotted against the metal radii (Figure 2a). In contrast, the longer M–B distances are consistent with κ^2^-BH_3_ for ^*i*^Pr-PDB complexes with smaller Er and Sm, but these shorten significantly for complexes with larger U and Nd consistent with a transition from κ^2^-BH_3_ to κ^3^-BH_3_ (Figure 2a). Averaging the four chelating M–B distances for each complex and plotting them against the ionic radius also reveals an excellent linear correlation (*R*^2^ = 0.998; Figure 2a).^21^ Plots of the individual chelating M–B distances show how these distances adjust to accommodate a change in the size of the metal (Figure 2b).

**Figure 3:**
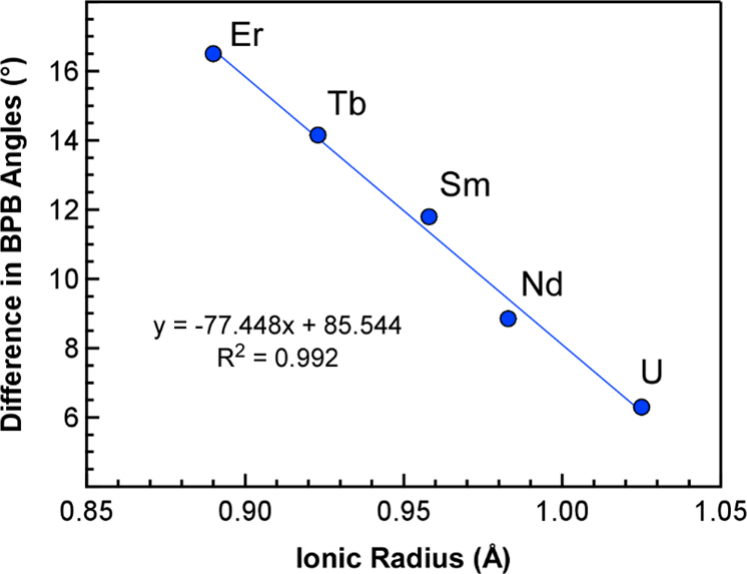
Difference in bridging and chelating B–P–B angles and in M(H_3_BP^*i*^Pr_2_BH_3_)_3_ (M-^*i*^Pr) complexes plotted as a function of metal radii.

As observed for the M–B distances, the size of the metal ion also has a significant influence on the bridging and chelating B–P–B angles. Bridging B–P–B angles in PDB complexes reported previously are larger than chelating B–P–B angles that bite down so the ligand can chelate to the metal.^16,20^ The same is true for the ^*i*^Pr-PDB and Et-PDB complexes reported here. The net difference in the larger bridging and smaller chelating B–P–B angles provides a parameter to quantify the inherent flexibility of the phosphinodiboranates and evaluate the influence of metal size on these angles. As shown in Figure 3, the difference between the bridging and average chelating B–P–B angles is the largest for the smallest metal in the series Er^3+^ (0.89 Å) at a difference of 16.5°, which emphasizes the remarkable flexibility of the ^*i*^Pr-PDB ligand. Plotting the difference in B–P–B angles against metal size reveals that the differences are highly correlated to metal size and decrease as the size of the metal ion increases from Er^3+^ (16.5°) to U^3+^ (6.3°).

**Figure 4:**
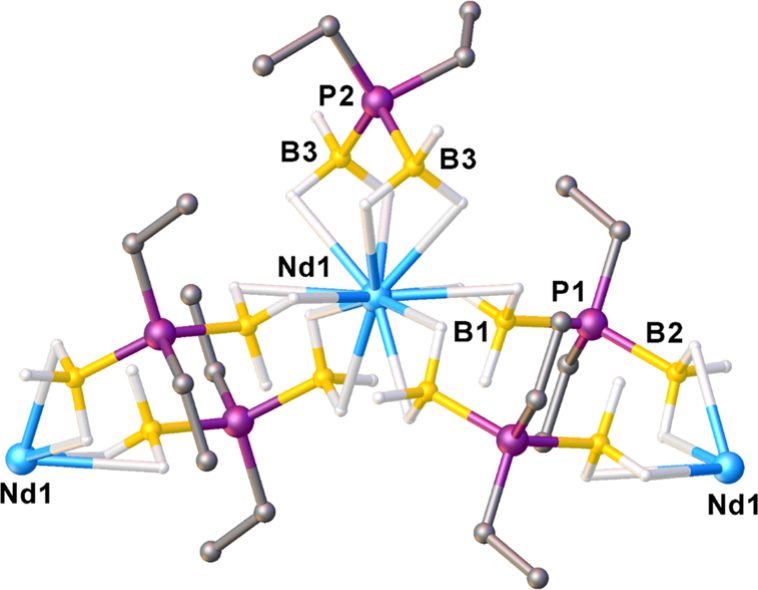
Polymeric structure of Nd(H_3_BPEt_2_BH_3_)_3_ (Nd-Et) from single-crystal XRD data. Hydrogen atoms attached to carbon and disordered components were omitted from the figure. Ball and stick representation shown for easier viewing of all the atoms.

U(H_3_BPEt_2_BH_3_)_3_ (U-Et) and Nd(H_3_BPEt_2_BH_3_)_3_ (Nd-Et) were prepared as described for the ^*i*^Pr-PDB complexes. Crystals of Nd-Et suitable for single-crystal XRD studies were isolated. Nd-Et has a polymeric structure that is markedly different compared to those of dimeric Nd-^*i*^Pr and Nd-^*t*^Bu (Figure 4). Each Nd^3+^ is coordinated by one chelating Et-PDB ligand and four bridging Et-PDB ligands. The chelating Nd–B distances are 2.933(6) Å, consistent with κ^2^-BH_3_, whereas the two bridging Nd–B distances are different: the shorter distance suggests κ^3^-BH_3_ (2.706(5) Å), whereas the longer distance suggests κ^2^-BH_3_ (2.871(5) Å). As with dimeric structures such as Nd-^*i*^Pr, the bridging B–P–B angle at 120.6(3)° is larger than the chelating B–P–B angle at 109.2(4)°, giving a net difference of 11.4°.

Crystals of U-Et were not suitable for XRD studies, but we suspect that it adopts the same structure as Nd-Et based on IR comparison (see below) and separate crystallographic studies with U(H_3_BPMe_2_BH_3_)_3_ (U-Me). Prior to our discovery that ball milling reactions improve the yield of f-element PDB complexes,^16^ we prepared U-Me in low yield by mixing UI_3_(1,4-dioxane)_1.5_ with 3 equiv of K(H_3_BPMe_2_BH_3_) in Et_2_O (Figure S4, Supporting Information). XRD analysis of the few crystals that managed to be isolated revealed U-Me to be isostructural with Nd-Et (both crystallize in the *C*2/*c* space group). As with Nd-Et, the bridging U–B distances are asymmetric at 2.709(10) and 2.896(9) Å and the chelating U–B distances are 2.918(8) Å, consistent with κ^2^-BH_3_ groups. The chelating and bridging B–P–B angles are 106.2(4)° and 118.6(4)°, respectively, to give a difference of 12.4°.

**Figure 5:**
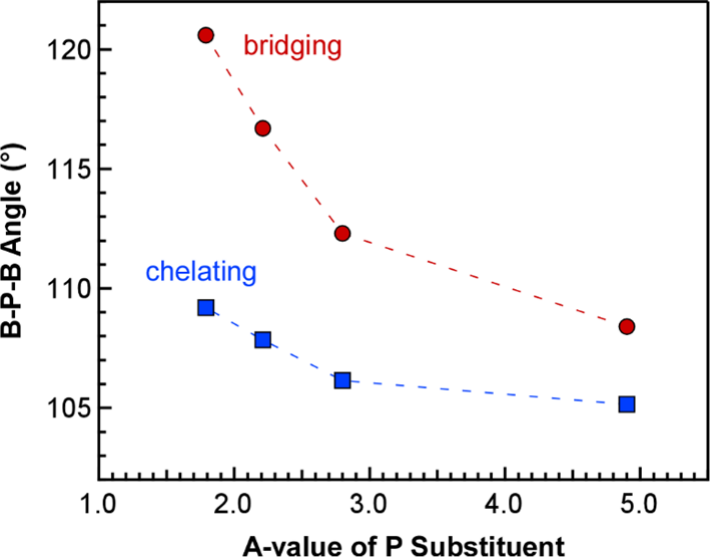
Plot of average bridging and chelating B–P–B angles of Nd(H_3_BPR_2_BH_3_)_3_ (R = Et, ^*i*^Pr, Ph, or ^*t*^Bu) obtained from single-crystal XRD studies vs the *A* values of the phosphorus substituents in kcal/mol.^26^

To better understand the influence of the phosphorus substituents on the structures of PDB complexes, we compared the bridging and chelating B–P–B angles of Nd-Et to those of Nd-^*i*^Pr described above and Nd-^*t*^Bu and Nd-Ph reported previously^16,20^ by plotting them against the *A* value of each substituent (Figure 5). *A* values are experimentally derived Gibbs free energies (in kcal·mol^–1^) associated with the axial/equatorial preference of cyclohexanes with differently sized substituents,^26^ and these values have long been used as a quantitative measure of steric bulk. *A* values decrease across the series in order of ^*t*^Bu (4.9) > Ph (2.8) > ^*i*^Pr (2.21) > Et (1.79).^26^ Consistently, both the bridging and chelating B–P–B angles are highly correlated to the *A* value of the phosphorus substituent and show a smooth decrease as the bulk increases from ethyl to *tert*-butyl. A similar trend is observed for the B–P–B angles in U complexes U-^*t*^Bu, U-Ph, and U-^*i*^Pr. These correlations indicate that increasing size and steric pressure of the phosphorus substituents decreases the flexibility of B–P–B angles in phosphinodiboranate ligands.

### Spectroscopic Analysis

**Figure 6:**
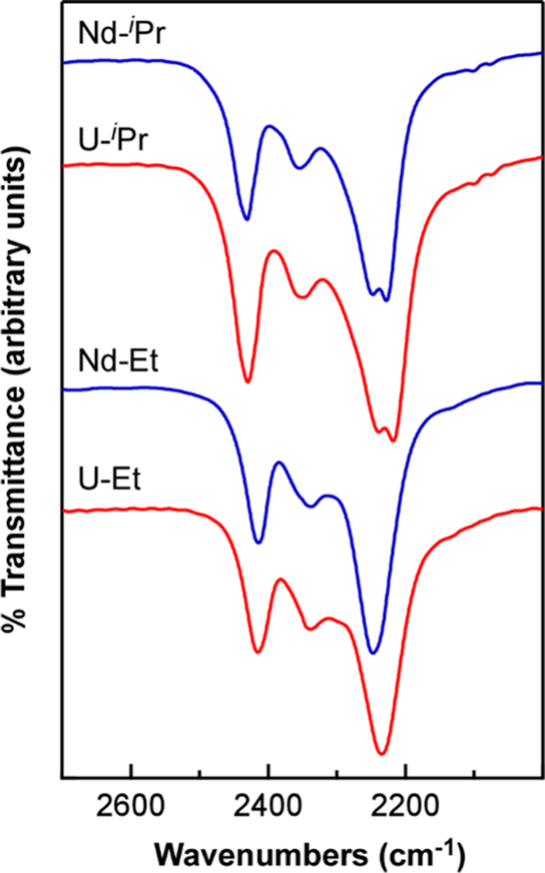
Comparison of solid-state infrared spectra (KBr) of U-^*i*^Pr and U-Et (red) and Nd-^*i*^Pr and Nd-Et (blue).

Infrared spectra collected on the new PDB complexes revealed the presence of terminal B–H and bridging B–H–M vibrational stretches between 2200 and 2500 cm^–1^ (Figure 6), as is typically observed for borohydride complexes.^27,28^ The spectrum of U-^*i*^Pr revealed four prominent B–H stretches at 2430, 2351, 2240, and 2218 cm^–1^. Nd-^*i*^Pr revealed an identical spectral profile with stretching absorptions at 2432, 2354, 2248, and 2228 cm^–1^. In contrast, the spectrum of U-Et revealed only three absorptions at 2414, 2339, and 2234 cm^–1^, but the profile again matched the spectrum of the corresponding Nd complex (2414, 2339, and 2248 cm^–1^) suggesting that U-Et and Nd-Et adopt similar solid-state structures. The terminal B–H stretches for each set of U and Nd complexes were identical within error, but the bridging B–H–M stretches for the U complexes were shifted to lower energy by 8–14 cm^–1^. This could be attributed to a slight increase in M–H–B covalency for M = U vs M = Nd, but these data do not allow us to rule out other potential explanations such as metal-size-dependent changes in structure and Lewis acidity. In this context, we note that the two B–H–M stretching absorptions observed for U-^*i*^Pr and Nd-^*i*^Pr merge to form a single feature as the series is traversed in the order Sm-^*i*^Pr, Tb-^*i*^Pr, and Er-^*i*^Pr (Figure S27, Supporting Information).

^1^H and ^11^B NMR data were collected on the M-^*i*^Pr and M-Et complexes to evaluate the effect of the phosphorus substituents on solution speciation and the degree of oligomerization. All five M-^*i*^Pr complexes revealed paramagnetically shifted ^1^H and ^11^B NMR resonances consistent with the presence of both a dimer and monomer in solution (Table 1). As with M-^*t*^Bu complexes, U-^*i*^Pr and Nd-^*i*^Pr with the largest metals showed the greatest ratio of dimer to monomer, whereas Tb-^*i*^Pr and Er-^*i*^Pr with the smallest metals in the series exist predominately as monomers. ^1^H and ^11^B NMR spectra collected on U-Et and Nd-Et again suggest both dimers and monomers, but the spectra indicate that the monomeric structures dominate in solution, contrasting observations made for U and Nd phosphinodiboranate complexes with larger phosphorus substituents.

**Table 1:** ^1^H and ^11^B NMR Resonances for the BH_3_ Groups in Each Complex in C_6_D_6_

	dimer		monomer		
complex	^1^H	^11^B	^1^H	^11^B	dimer/monomer molar ratio^a^
U-^*i*^Pr	74.2, 91.8	128.2, 298.1	95.1	186.6	0.88
Nd-^*i*^Pr	76.3, 77.1	69.9, 165.2	82.1	91.2	0.39
Sm-^*i*^Pr	–2.61, −1.74	–31.0^b^	–3.67	–33.9	0.31
Tb-^*i*^Pr	not observed	–741.6, −502.2	–356.9	–546.5	0.02^c^
Er-^*i*^Pr	not observed	–427.7, −234.3	–182.0	–270.5	0.02^c^
U-Et	72.9, 86.6	140.1^b^	89.5	192.5	0.04
Nd-Et	75.1, 76.9	73.5, 151.2	81.0	90.0	0.03

a Based on ^1^H NMR integrations measured of the BH_3_ resonances at 20 °C unless stated otherwise.

b Second resonance unresolved or too weak to observe.

c Based on ^11^B NMR integrations at 20 °C.

Previous variable-temperature NMR studies of M-^*t*^Bu complexes in C_6_D_6_ revealed measurable differences in the dimer/monomer equilibrium for U^3+^ as compared with similarly sized lanthanide ions like La^3+^ and Ce^3+^. Thermodynamic values obtained from Van’t Hoff plots revealed ∼1 kcal/mol increase in the Δ*H* and Δ*G* for M = U, which was attributed to increased covalency in the U–H–B bonds.^21^ Unfortunately, NMR data collected on U-^*i*^Pr and Nd-^*i*^Pr revealed that ^*i*^Pr-PDB complexes are not as amenable to teasing out such small differences in the thermodynamic values (though DFT calculations were used to quantify these values; see below). NMR spectra for U-^*i*^Pr and Nd-^*i*^Pr showed additional resonances in the baseline that were not observed in the spectra of the ^*t*^Bu-PDB complexes. These resonances were concentration dependent and appeared most prominently in saturated solutions, and we suspect that they are likely attributed to higher order oligomers (e.g., trimer, tetramer). This hypothesis is consistent with the increased flexibility in the B–P–B angle for the ^*i*^Pr-PDB ligand that allow a wider range of structures to be adopted when compared to those with ^*t*^Bu-PDB.

Though exact quantitative concentrations were not obtained from the NMR solutions, as needed for calculating equilibrium constants, we found it informative to conduct a coarse grain comparison of the dimer/monomer ratios to determine how the ratios change in response to PDB substituents and metal size. This analysis is possible because the PDB complexes have similar solubility in C_6_D_6_ (<5 mg/mL), and the dimers and monomers appear to be the major species present in solution. It is worth noting here that the two-to-one ratio of chelating-to-bridging PDB ligand resonances assigned as dimers could also be consistent with higher order oligomers that give the same ratio, similar to that reported by Mirkin and co-workers for dinuclear and tetranuclear Rh complexes.^29^ We have so far been unable to rule out the possibility of these higher order oligomers for some of the phosphinodiboranate complexes (preliminary DOSY experiments were unsuccessful), but they appear unlikely, given that the dimers are isolated almost exclusively in the solid state. Moreover, our calculations described previously and below consistently suggest that dimers should be the dominant oligomer in solution. The only potential exception observed thus far is the Et-PDB complexes, as discussed in the following section (*vide infra*). However, given that these complexes appear to exist almost exclusively as monomers in solution on the NMR time scale, the identity of the oligomer should have little bearing on the analysis that follows.

**Figure 7:**
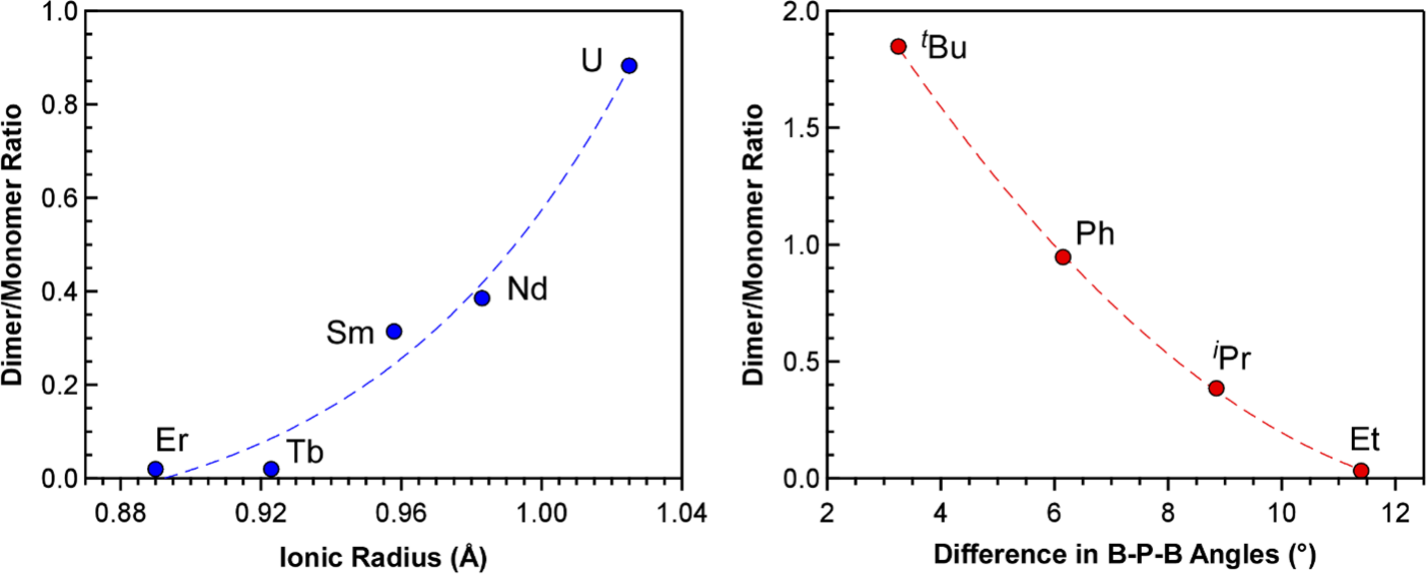
Left: Comparison of dimer/monomer ratios observed in the ^1^H NMR spectra of M(H_3_BP^*i*^Pr_2_BH_3_)_3_ (M-^*i*^Pr) complexes in C_6_D_6_ plotted as a function of ionic radii of the metal (M = U, Nd, Sm, Tb, and Er; CN = 6).^22^ Right: Comparison of dimer/monomer ratios observed in the ^1^H NMR spectra of Nd(H_3_BPR_2_BH_3_)_3_ complexes in C_6_D_6_ (R = ^*t*^Bu, Ph, ^*i*^Pr, Et) plotted as a function of the difference in bridging and average chelating B–P–B angle.

We first compared the molar dimer/monomer ratio of the five ^*i*^Pr-PDB complexes to their respective metal radii (Figure 7). As expected, the plot revealed a relatively smooth increase as the size of the trivalent metal radii increased from Er^3+^ to U^3+^.

Next, we compared data for the neodymium complexes with Et-PDB and ^*i*^Pr-PDB to those reported previously with ^*t*^Bu-PDB and Ph-PDB to determine the influence of the substituents attached to phosphorus. The solid-state structures of all four Nd(H_3_BPR_2_BH_3_)_3_ complexes are known,^16,20^ which allowed their dimer/monomer molar ratios obtained from NMR spectroscopy to be plotted against the difference in the chelating and bridging B–P–B angles from single-crystal XRD (Figure 7). The plot shows that the dimer/monomer ratio in solution is highly correlated to the substituent-dependent differences in B–P–B angles observed in the solid state.

**Figure 8:**
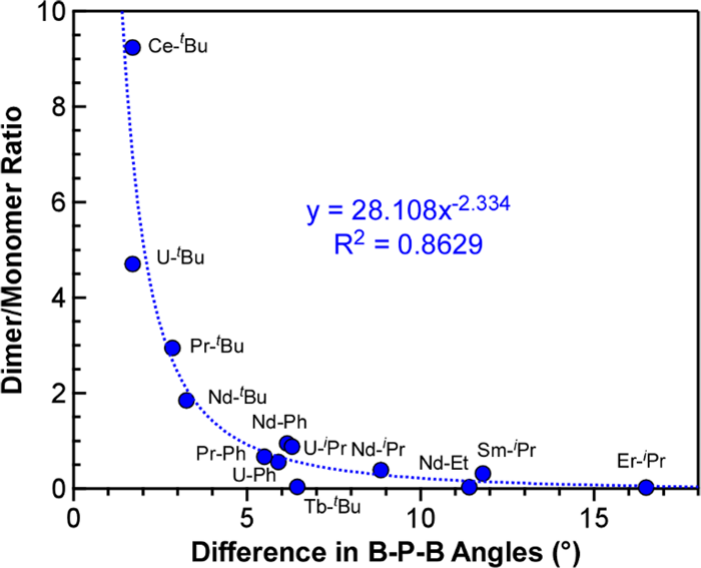
Comparison of dimer/monomer ratios observed in the ^1^H NMR spectra of all crystallographically characterized M(H_3_BPR_2_BH_3_)_3_ complexes in C_6_D_6_ plotted as a function of the absolute difference in bridging and average chelating B–P–B angles.

Collectively, the plots in Figure 7 suggested that the degree of solution oligomerization, as measured by NMR spectroscopy, can be modeled using the difference in the bridging and average chelating B–P–B angle, a parameter that takes into account both the size of the metal and the size of the phosphorus substituent. Plotting all of the available data reported here and published previously supports this hypothesis (Figure 8).^16,20^ The data can be fit to a power law showing how the ratio of dimer to monomer increases and approaches infinity as the difference in the B–P–B angles goes to zero. These results suggest that the rigidity of the B–P–B angle, which can be tuned via the size and steric pressure of the phosphorus substituents, controls the extent of dimerization in solution with different trivalent f-metals.

### Density Functional Theory (DFT) Calculations

DFT calculations were performed to further evaluate the structures of lanthanide and uranium PDB complexes and their comparative energies. Calculations allowed structures with differing degrees of oligomerization to be determined (including those not isolable experimentally) for comparison to the experimental results. Gas phase geometry optimization and harmonic vibrational analysis were performed using the TPSS-D3 functional and a basis set of near triple-ζ quality (see Experimental Section for details). When a solid-state structure was available, the geometry optimization was started from the coordinates obtained from the experiment.

The first series of calculations focused on the impact of the ligand and metal on the molecular geometry of the dimer. Though Nd-Et is a polymeric structure in the solid state, this complex was initially modeled as a dimer so that structural comparisons could be made with other dimeric Nd complexes with different substituents attached to phosphorus (Figure S28, Supporting Information). A similar choice was made for U-Et where no solid-state structure was obtained. Following the initial DFT optimizations from the solid-state structures, an extensive conformational search was performed using a computationally efficient method (specifically the GFN2-xTB tight binding approach) using the CREST algorithm^30^ for the Nd-Et, Nd-^*i*^Pr, Nd-Ph, and Nd-^*t*^Bu dimers. The search resulted in 245, 114, 86, and 138 conformers within 6 kcal/mol at the GFN2-xTB level. The lowest energy conformer predicted by this lower level of theory was subsequently reoptimized with DFT (TPSS-D3). No conformational search could be performed with xTB methods for the U species since DFTB parameters are not available. Calculations involving U were computed by starting from the DFT optimized geometries obtained with Nd. Analogous conformational searches were performed for the other dimers studied (Er-^*i*^Pr, Tb-^*i*^Pr, and Sm-^*i*^Pr).

**Figure 9:**
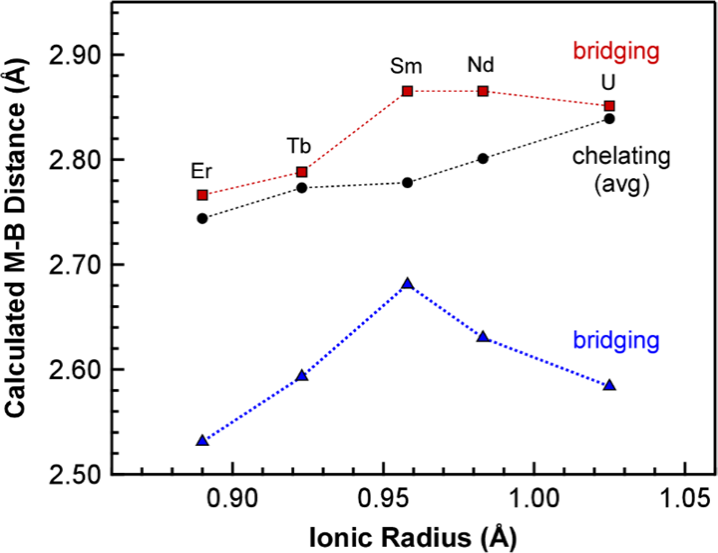
Plot of the DFT (TPSS-D3) calculated M–B distances in M(H_3_BP^*i*^Pr_2_BH_3_)_3_ vs the ionic radius of the corresponding metal (M = U, Nd, Sm, Tb, Er; CN = 6.).

Calculated structures for the ^*i*^Pr-PDB dimers reproduced the most prominent structural features in the experimental data. All of the ^*i*^Pr-PDB complexes showed the observed binding asymmetry in the bridging PDB ligands with one short M–B distance assigned as κ^3^-BH_3_ and one long M–B distance assigned as κ^2^-BH_3_. The chelating M–B distances also revealed a linear increase as the ionic radius of the metal increased (Figure 9, Table 2). The only significant distinction between the experimental and calculated structures is that one set of bridging M–B distances in the calculated structures are slightly longer than the chelating M–B distances (but still within the range for κ^2^-BH_3_). Subtle differences in the linearity of the plotted data are also observed when comparing experimental and calculated plots of the shorter bridging M–B distances vs ionic radii. Collectively, these differences likely reflect small energy differences between various structural conformers. They could also indicate experimental solid-state packing effects not captured in the calculated data, as shown for other complexes with chelating borohydrides.^31^ The difference between chelating and bridging B–P–B angles was correlated with the ionic radii of the metals for the ^*i*^Pr-PDB complexes consistent with experimental results (Figure 3). The difference was higher for Er-^*i*^Pr (14.6°) and decreased to 8.6° for Nd-^*i*^Pr. For U-^*i*^Pr, the difference was slightly higher (11.4°) than that for Nd-^*i*^Pr.

**Table 2:** Selected Bond Distances (Å) and Ligand B–P–B Angles (deg) from the DFT (TPSS-D3) Optimized Structures of the Dimers

complex	M–B (Å) chelating	M–B (Å) bridging		chelating angle	bridging angle	Δ angle
U-^*t*^Bu	2.846	2.599	2.754	104.9	113.6	8.7
U-Ph	2.807	2.574	2.837	109.9	118.1	8.2
U-^*i*^Pr	2.839	2.584	2.851	106.4	117.8	11.4
U-Et	2.787	2.685	2.900	110.9	118.6	7.6
Nd-^*t*^Bu	2.820	2.607	2.800	107.1	117.4	10.3
Nd-Ph	2.796	2.600	2.826	111.0	117.7	6.8
Nd-^*i*^Pr	2.801	2.630	2.865	110.1	118.7	8.6
Nd-Et	2.793	2.672	2.874	110.7	119.2	8.5
Sm-^*i*^Pr	2.778	2.681	2.865	110.5	116.9	6.4
Tb-^*i*^Pr	2.773	2.593	2.788	108.3	121.5	13.1
Er-^*i*^Pr	2.744	2.531	2.766	103.5	118.1	14.6

We next compared the influence of R substituents on the structures of the calculated dimers. The average chelating U–B bond distances decreased in the order ^*t*^Bu > ^*i*^Pr > Ph > Et; the longest average chelating distance of 2.846 Å for U-^*t*^Bu decreased to 2.787 Å for U-Et to give a net difference of 0.059 Å across the series. The Nd complexes showed the same trend, decreasing in the order ^*t*^Bu > ^*i*^Pr > Ph > Et, but the net difference between largest and smallest average chelating Nd–B distances was smaller than for U (Δ = 0.027 vs 0.059 Å). By comparison, the bridging Nd–B and U–B distances showed more variation and the relative ordering depended on the bridging bond distance in question. For example, the shorter bridging Nd–B distances decreased from 2.672 Å in Nd-Et to 2.600 Å in Nd-Ph, whereas the longer bridging Nd–B distances decreased from 2.874 Å in Nd-Et to 2.800 Å in Nd-^*t*^Bu. Similar ordering was observed for the bridging U–B distances, although with greater variation in the bond distances (Δ = 0.111 and 0.146 Å) when compared to those in the Nd complexes (Δ = 0.072 and 0.074 Å).

**Figure 10:**
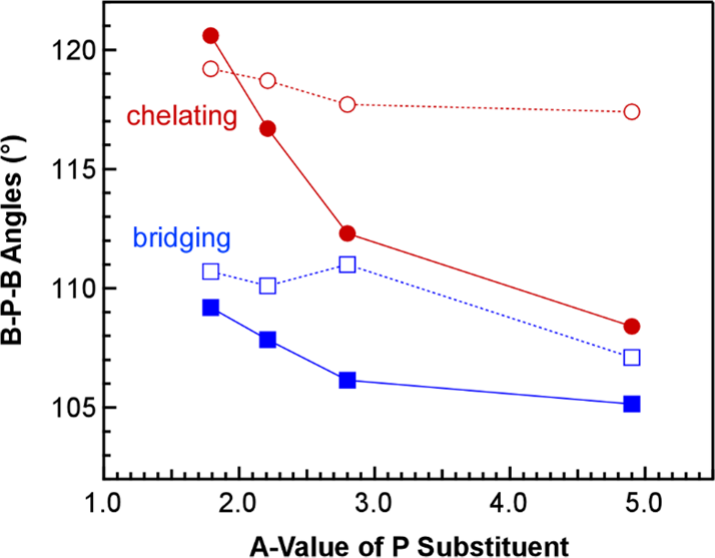
Comparison of B–P–B angles for Nd(H_3_BPR_2_BH_3_)_3_ plotted against the *A* values of the phosphorus substituents (given in kcal/mol). Experimental B–P–B angles are shown as solid lines and markers, and calculated angles are shown as dotted lines and open markers.

As observed in comparisons of the calculated ^*i*^Pr-PDB structures with different metals, the bridging and chelating B–P–B angles showed the same substituent-dependent trends as the experimental results, as exemplified with the *A* value plots for the Nd complexes in Figure 10. The magnitude of the calculated B–P–B angle differences, however, was generally smaller and had a few outliers. We note that the calculated angles represent those in the lowest energy structure obtained from the conformational search, and these could certainly be different in the solid state and solution, given the relatively small energy differences between multiple low energy conformers. In this context, we note that crystal packing and the associated intermolecular interactions are not included in the gas phase DFT calculations. Despite these differences, the gas phase calculations reproduce the general experimental trends and corroborate how B–P–B angles in PDB complexes are quite sensitive to the metal identity and phosphorus substituent size.

Although experimentally obtained thermodynamic data were not collected (*vide supra*), Gibbs free energies, enthalpies, and entropies of reaction were calculated using DFT (TPSS-D3) for M_2_(H_3_BPR_2_BH_3_)_6_ → 2 M(H_3_BPR_2_BH_3_)_3_ (R = Et, ^*i*^Pr, Ph and ^*t*^Bu; Table S7, Supporting Information). Solvent effects were included by performing a single point calculation with the COSMO model^32^ for benzene on the gas phase structure. For the ^*i*^Pr-PDB complexes, the Gibbs free energy of the reaction tended to increase from the smallest metals Tb and Er to U, indicating that the dimer is more favorable for the larger ionic radii metals, consistent with the experiment. In contrast, Gibbs free energy and enthalpy calculations with different R groups and the same metal (Nd or U) were less consistent with the experimental dimer/monomer ratios observed by NMR spectroscopy (Table 3). U-Et and Nd-Et had the lowest calculated Δ*G* and Δ*H* values, in alignment with the greater experimental preference for monomers, but the values for U-Ph and Nd-Ph were calculated to be several kilocalories per mole larger than those for U-^*t*^Bu and Nd-^*t*^Bu with bulkier *tert*-butyl substituents. Moreover, the calculated values for U-^*i*^Pr are also slightly higher than those for U-^*t*^Bu, whereas Nd-^*i*^Pr and Nd-^*t*^Bu follow the experimental trend. These small discrepancies are likely attributed to different rotational conformations of the phenyl and isopropyl substituents, which are less isotropic compared to *tert*-butyl substituents. This is relevant given that the experimental dimer/monomer ratios represent a weighted average of deoligomerization energies for all of the conformational isomers present in solution, whereas the calculations in Table 3 only capture energies associated with a single set of isomers.

**Table 3:** Thermochemical Data at 298.15 K for the TPSS-D3 Free Energies, Enthalpies, and Entropies for Reaction Dimer → 2 Monomer^a^

complex	Δ*G* (kcal·mol^–1^)	Δ*H* (kcal·mol^–1^)	Δ*S* (kcal·mol^–1^·K^–1^)
U-Ph	9.2	24.8	0.059
Nd-Ph	8.7	24.1	0.058
U-^*i*^Pr	8.7	23.6	0.057
U-^*t*^Bu	7.1	22.0	0.057
Nd-^*t*^Bu	6.6	20.7	0.054
U-Et	3.8	18.5	0.056
Nd-^*i*^Pr	3.6	18.1	0.055
Sm-^*i*^Pr	3.5	18.3	0.056
Nd-Et	1.8	16.0	0.054
Er-^*i*^Pr	1.0	15.7	0.056
Tb-^*i*^Pr	–0.2	14.6	0.056

a Free energies have been computed by assuming a concentration of 1 M for all species taking benzene as the solvent.

**Figure 11:**
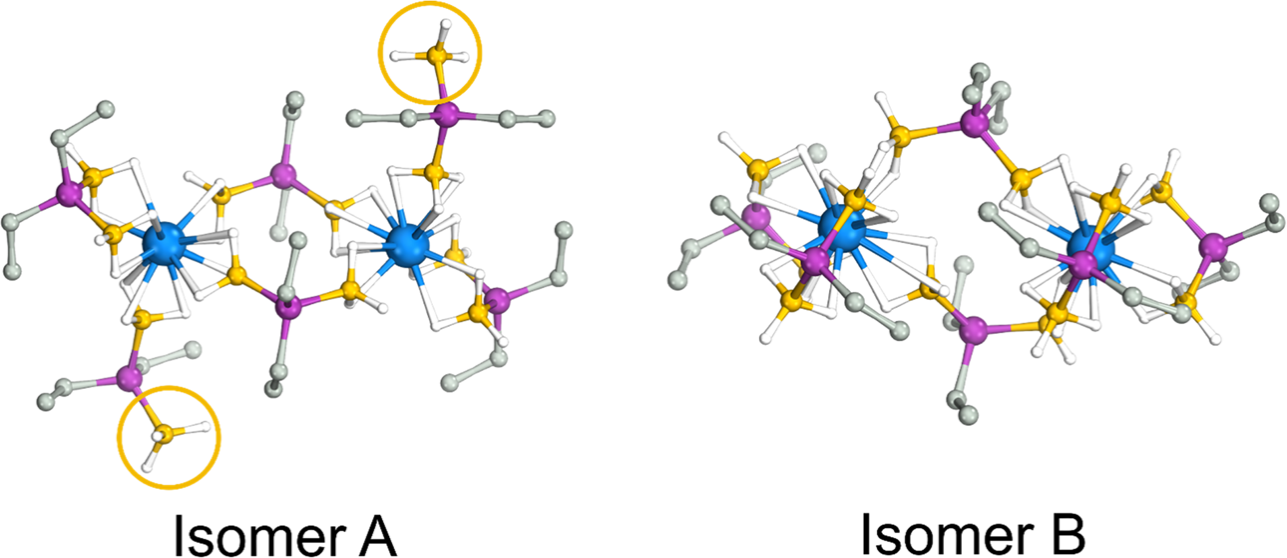
DFT optimized dimers of Nd-Et with different ligand coordination modes for Et-PDB. The orange circles highlight the dangling BH_3_ groups. Hydrogen atoms attached to carbon have been omitted for clarity.

Following the DFT study of the dimers, further computations were performed on Nd-Et. Recall that the experimental structure of Nd-Et is polymeric, with one of the PDB ligands in the typical dimer motif bridging to adjacent metals. NMR data suggested that Nd-Et dissolves to form the usual mixture of monomers and dimers, as observed for other PDB complexes, but the possibility that the bridging BH_3_ group observed in the polymer remains unbound in solution to form a dimer with a “dangling” BH_3_ group was also considered. These “dangling” groups have been seen in the solid-state structure of other PDB complexes,^16,17,21^ but only in the presence of competing donor ligands like THF. Indeed, as described in our previous reports, similar attempts to crystallize Nd-Et in the presence of THF resulted in adventitious crystals of Nd(H_3_BPEt_2_BH_3_)_3_(THF)_3_ with dangling BH_3_ groups (Figure S5, Supporting Information). Species with dangling BH_3_ groups are also possible as part of the deoligomerization process and may be found along the reaction coordinate diagram. To evaluate the energy of these putative species, we performed calculations on the Nd-Et dimer with and without dangling BH_3_ groups to compare their energies (isomers A and B, respectively, in Figure 11). The calculation revealed that isomer B is the energetically more stable isomer; isomer A has a higher free energy of 24.9 kcal/mol. This supports the hypothesis that deoligomerization of the solid-state polymer occurs when Nd-Et is dissolved to form mixtures of monomers and dimers (or higher order oligomers) with all BH_3_ groups bound. Calculations on U-Et yielded similar results, with isomer B being favored by 22.7 kcal/mol in Gibbs’s energy. These results suggest that species containing dangling BH_3_ groups are unlikely to persist in homoleptic PDB complexes containing these metals.

**Scheme 4:**
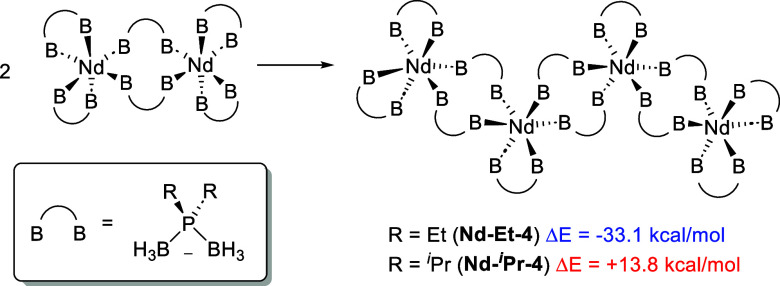
General Structures and Calculated Energies Associated with the Formation of Nd-Et-4 and Nd-^*i*^Pr-4 from Their Corresponding Dimers Δ*E* is shown instead of Δ*G* because the vibrational frequencies were not calculated for Nd-^*i*^Pr-4 due to the relatively large system size.

To further evaluate the structure in the solid state, computations were performed with a tetrameric oligomer (Nd-Et-4) as a model system of the solid (Scheme 4). Starting geometries were taken from the experimental polymeric Nd-Et structure (Figure S2). Note that a smaller basis set (def2-SVP) was used on light atoms due to the size of the tetramer. Given the prior results on the dimer, the ligands at the capping ends of the tetramer were truncated with chelating ligands since this is expected to be more favorable than leaving residual dangling bonds. A hypothetical oligomer, Nd-^*i*^Pr-4, was also built by replacing Et-PDB ligands by ^*i*^Pr-PDB ligands. This structure was optimized with DFT to determine how the Nd–B distances and B–P–B angles change as a function of ligand choice in the larger model. Despite the differences between the polymeric experimental structure for Nd-Et and the calculated tetrameric structure of Nd-Et-4, the calculated and experimental bond distances and angles associated with the chelating and bridging Et-PDB bond distances are in relatively good agreement (see Supporting Information).

Calculations for the formation of the tetramers Nd-Et-4 and Nd-^*i*^Pr-4 from their corresponding dimers appear to account for the difference in solid-state structures of Nd-Et and Nd-^*i*^Pr (Scheme 4). In the case of Nd-Et, the free energy of the reaction is exergonic at −10.7 kcal/mol (an electronic energy difference of −33.1 kcal/mol), supporting that the formation of a tetramer is favorable for this system. While vibrational frequencies were not computed for Nd-^*i*^Pr-4 due to system size, the formation of this tetramer is uphill by +13.8 kcal/mol in electronic energy. Overall, these results are fully consistent with experimental observations that Nd-Et exists as an extended coordination polymer in the solid state, whereas Nd-^*i*^Pr exists as a dimer. Moreover, they confirm how relatively subtle changes in the steric properties of phosphorus substituents can have a remarkable influence on the structure of phosphinodiboranate complexes.

## Conclusions

In summary, we described the synthesis, structures, and solution speciation of homoleptic lanthanide and uranium phosphinodiboranates with isopropyl and ethyl substituents. Single-crystal XRD studies of the M(H_3_BP^*i*^Pr_2_BH_3_)_3_ complexes with M = U, Nd, Sm, Tb, and Er revealed that they crystallize as dimers, whereas Nd(H_3_BPEt_2_BH_3_)_3_ and U(H_3_BPMe_2_BH_3_)_3_ with smaller alkyl substituents crystallize as coordination polymers. Comparisons to M(H_3_BP^*t*^Bu_2_BH_3_)_3_ and M(H_3_BPPh_2_BH_3_)_3_ complexes reported previously show how the sizes of the metal and phosphorus substituents have a significant influence on the solution and solid-state structures. The stepwise decrease in steric bulk from R = ^*t*^Bu to R = Et yields increased flexibility in the phosphinodiboranate B–P–B angles, as is evident by measured differences in chelating and bridging B–P–B angles when the identity of the metal is held constant. Moreover, decreasing the size of the metal in ^*i*^Pr-PDB complexes causes larger differences in chelating B–P–B angles that correlate to a decreasing ratio of dimer to monomer when the complexes are dissolved in benzene. The combined influence of both the phosphorus substituent size and metal radius on the preferred solution speciation of all known M(H_3_BPR_2_BH_3_)_3_ complexes can be modeled effectively using the difference in bridging and chelating B–P–B angles in the experimental structures. DFT calculations reproduce the general experimental trends and showed that there is a relatively flat energy surface between many different conformers that likely contribute to experimentally observed differences in solution speciation. Free energy calculations comparing tetrameric and dimeric species account for the preferred adoption of polymeric and dimeric structures observed experimentally for Nd-Et and Nd-^*i*^Pr.

Overall, these results demonstrate how steric changes to phosphorus substituents relatively far removed from metal coordination sites can have a significant influence on solution speciation, deoligomerization energies, and the solid-state structure of PDB complexes with trivalent f-metals. Though none of the phosphinodiboranate complexes reported here are appreciably volatile, these insights are important given that depolymerization energies are known to play a governing influence on the volatility in other f-element borohydride complexes,^33^ including those containing aminodiboranates (the nitrogen congeners of phosphinodiboranates).^31,34−38^ Moreover, the results may also have important implications for disubstituted diorganophosphinates (X_2_PR_2_^–^) containing metal-donor groups other than X = BH_3_ (e.g., CR_2_, NR, O, S, and Se), especially those that have proven to be effective for trivalent f-element separations like dithiophosphinates (X = S).^39^ Steric-induced changes in the solution speciation of phosphinate complexes would be expected to influence their solvent extraction properties. In this context, the underlying origin of how different phosphorus substituents influence the selectivity of dithiophosphinate extractants (including those with differently sized alkyl substituents) continues to be a subject of debate.^39^

## Experimental Section

### General Considerations

All reactions were carried out under an atmosphere of N_2_ or Ar using glovebox or standard Schlenk techniques. All glassware was heated at 150 °C for at least 2 h and allowed to cool under a vacuum before use. Solvents were dried and deoxygenated using a Pure Process Technologies Solvent Purification System and stored over 3 Å molecular sieves. Deuterated solvents were deoxygenated on the Schlenk line by three freeze–pump–thaw cycles and stored over 3 Å molecular sieves for at least 3 days before use. K(H_3_BP^*i*^Pr_2_BH_3_), K(H_3_BPEt_2_BH_3_), and K(H_3_BPMe_2_BH_3_) were prepared as described previously for K(H_3_BPPh_2_BH_3_) and K(H_3_BP^*t*^Bu_2_BH_3_).^6,7^ UI_3_(THF)_4_ was prepared as described previously from UCl_4_.^40^ Anhydrous LnI_3_ salts were purchased in their highest purity from Alfa Aesar or Strem Chemicals and used as received.

^1^H NMR data were collected on a Bruker AVANCE-400 operating at 400 MHz or a Bruker AVANCE-500 operating at 500 MHz. The ^11^B NMR data were collected on a Bruker AVANCE-400 operating at 128 MHz or a Bruker AVANCE-500 operating at 160 MHz. Chemical shifts in C_6_D_6_ are reported in δ units relative to those in C_6_D_5_H (^1^H; δ 7.16 ppm) and BF_3_·Et_2_O (^11^B; δ 0.0 ppm). ^31^P NMR data were not collected because these resonances are typically too broad to be observed for paramagnetic phosphinodiboranate complexes due to paramagnetic broadening and coupling with the quadrupolar ^10^B and ^11^B nuclei. Microanalytical data (CHN) were collected using an EAI CE-440 elemental analyzer at the University of Iowa’s Shared Instrumentation Facility. IR spectra were acquired with a Thermo Scientific Nicolet iS5 in a N_2_-filled glovebox as KBr pellets. Mechanochemical reactions were carried out on a Form-Tech Scientific (FTS) FTS1000 shaker mill or a FlackTek SpeedMixer with a Teflon insert designed to accommodate FTS grinding jars. All mechanochemical reactions were conducted in 5 mL stainless steel “SmartSnap” (hermetic seal) grinding jars from FTS using two 5 mm stainless steel balls (304 grade) for grinding. Melting points were collected in sealed capillaries using a REACH melting point apparatus.

### Tris(diisopropylphosphinodiboranato)neodymium(III), Nd(H_3_BP^*i*^Pr_2_BH_3_)_3_ (Nd-^*i*^Pr)

NdI_3_ (0.100 g, 0.190 mmol) and K(H_3_BP^i^Pr_2_BH_3_) (0.105 g, 0.571 mmol) were loaded into a 5 mL FTS ball-milling jar with two 5 mm stainless steel balls and a few drops of pentane as a wetting solvent. The jars were hermetically sealed with electrical tape, transferred to a FlackTek SpeedMixer, and milled three times at 1800 rpm for 5 min each. The jar was transferred to a glovebox and opened to reveal a light blue paste. The crude mixture was suspended in Et_2_O, filtered, and evaporated to dryness under a vacuum to afford a light blue oil. The product was dissolved in a minimum amount of Et_2_O and stored in a freezer at −30 °C. Small, light blue blocks formed after 2 days. Yield: 62.4 mg (57%). Mp: 150 °C. Anal. calcd for C_18_H_60_B_6_P_3_Nd: C, 37.36; H, 10.45. Found: C, 36.95; H, 10.61. ^1^H NMR (500 MHz, C_6_D_6_, δ): 1.12 (br s, CH(C*H*_3_)_2_), 1.60 (br s, CH(C*H*_3_)_2_), 1.78 (br s, CH(C*H*_3_)_2_), 2.43 (br s, C*H*(CH_3_)_2_), 3.28 (br s, C*H*(CH_3_)_2_), 4.88 (br s, C*H*(CH_3_)_2_), 76.3 (br s, BH_3_, dimer), 77.1 (br s, BH_3_, dimer), 82.1 (br s, BH_3_, monomer). ^11^B NMR (160 MHz, C_6_D_6_, δ): 69.9 (br s, BH_3_, dimer), 91.2 (br s, BH_3_, monomer), 165.2 (br s, BH_3_, dimer). IR (KBr) ν̅_max_ (cm^–1^): 2961 (s), 2931 (m), 2896 (w), 2871 (m), 2432 (vs), 2354 (s), 2248 (vs), 2228 (vs), 1462 (s), 1386 (m), 1382 (w), 1233 (s), 1159 (w), 1062 (s), 1036 (m), 929 (w), 884 (m), 799 (m), 727 (m), 679 (s).

### Tris(diisopropylphosphinodiboranato)uranium(III), U(H_3_BP^*i*^Pr_2_BH_3_)_3_ (U-^*i*^Pr)

Prepared as described for Nd-^*i*^Pr with UI_3_(THF)_4_ (0.100 g, 0.110 mmol) and K(H_3_BP^*i*^Pr_2_BH_3_) (0.061 g, 0.330 mmol). Yield: 17.3 mg (23%). M.p.: 150 °C (dec). Anal. calcd for C_18_H_60_B_6_P_3_U: C, 32.15; H, 8.99. Found: C, 32.43; H, 8.50. ^1^H NMR (500 MHz, C_6_D_6_, δ): 0.94 (br s, CH(C*H*_3_)_2_), 1.05 (br s, CH(C*H*_3_)_2_), 2.30 (br s, C*H*(CH_3_)_2_), 2.73 (br s, C*H*(CH_3_)_2_), 3.95 (br s, C*H*(CH_3_)_2_), 74.2 (br s, BH_3_, dimer), 91.8 (br s, BH_3_, dimer), 95.1 (br s, BH_3_, monomer). ^11^B NMR (160 MHz, C_6_D_6_, δ): 128.2 (br s, dimer, chelating), 186.6 (br s, monomer), 298.1 (br s, dimer, bridging). IR (KBr) ν̅_max_ (cm^–1^): 2959 (s), 2932 (s), 2894 (s), 2871 (s), 2430 (vs), 2351 (s), 2240 (vs), 2218 (vs), 1462 (s), 1384 (m), 1367 (m), 1231 (s), 1183 (w), 1159 (w), 1131 (w), 1102 (w), 1060 (s), 1034 (s), 927 (w), 884 (m), 795 (m), 727 (s), 677 (s).

### Tris(diethylphosphinodiboranato)uranium(III), U(H_3_BPEt_2_BH_3_)_3_ (U-Et)

Prepared as described for Nd-^*i*^Pr with UI_3_(THF)_4_ (0.100 g, 0.110 mmol) and K(H_3_BPEt_2_BH_3_) (0.052 g, 0.330 mmol). Yield: 14.8 mg (18%). ^1^H NMR (500 MHz, C_6_D_6_, δ): 0.22 (br s, CH_2_C*H*_3_, monomer), 0.43 (br s, C*H*_2_CH_3_, monomer), 1.50 (br s, CH_2_CH_3_, dimer), 1.58 (br s, CH_2_CH_3_, dimer), 3.60 (br s, CH_2_CH_3_, dimer), 72.9 (br s, BH_3_, dimer, chelating), 86.6 (br s, BH_3_ dimer, bridging), 89.5 (br s, BH_3_, monomer). ^11^B NMR (160 MHz, C_6_D_6_, δ): 140.1 (br s, dimer); 192.5 (br s, monomer). IR (KBr) ν̅_max_ (cm^–1^): 2971 (s), 2938 (s), 2911 (m), 2880 (m), 2414 (vs), 2339 (s), 2234 (vs), 1455 (m), 1413 (w), 1379 (w), 1258 (m), 1219 (s), 1174 (s), 1128 (s), 1071 (s), 1037 (s), 1013 (s), 785 (s), 741 (w), 679 (s).

### Tris(diethylphosphinodiboranato)neodymium(III), Nd(H_3_BPEt_2_BH_3_)_3_ (Nd-Et)

Prepared as described for Nd-^*i*^Pr with NdI_3_ (0.100 g, 0.110 mmol) and K(H_3_BPEt_2_BH_3_) (0.061 g, 0.330 mmol). Yield: 17.3 mg (23%). Anal. calcd for C_12_H_48_B_6_P_3_Nd: C, 29.14; H, 9.78. Found: C, 28.70; H, 9.44. ^1^H NMR (500 MHz, C_6_D_6_, δ): 0.68 (br s, CH_2_CH_3_, dimer), 1.20 (br s, CH_2_C*H*_3_, monomer), 1.54 (br s, CH_2_CH_3_, dimer), 1.84 (br s, CH_2_CH_3_, dimer), 2.08 (br s, C*H*_2_CH_3_, monomer), 3.88 (br s, CH_2_CH_3_, dimer), 75.1 (br s, BH_3_, dimer, bridging), 76.9 (br s, BH_3_, dimer, chelating), 81.0 (br s, BH_3_, monomer). ^11^B NMR (160 MHz, C_6_D_6_, δ): 73.5 (br s, dimer), 90.0 (br s, monomer), 151.2 (br s dimer). IR (KBr) ν̅_max_ (cm^–1^): 2972 (s), 2938 (s), 2911 (m), 2879 (m), 2414 (vs), 2339 (s), 2248 (vs), 1456 (s), 1412 (m), 1379 (m), 1260 (w), 1222 (s), 1177 (m), 1129 (m), 1071 (s), 1036 (s), 1033 (s), 786 (s), 737 (w), 676 (s).

### Tris(diisopropylphosphinodiboranato)samarium(III), Sm(H_3_BP^*i*^Pr_2_BH_3_)_3_ (Sm-^*i*^Pr)

Prepared as described for Er-^*i*^Pr using SmBr_3_ (0.103 g, 0.264 mmol) and K(H_3_BP^*i*^Pr_2_BH_3_) (0.142 g, 0.772 mmol). Vapor diffusion with DCM and pentane at −30 °C yielded small clear blocks that were isolated after approximately 20 days. Yield: 52.2 mg (34%). ^1^H NMR (400 MHz, C_6_D_6_, δ): −3.67 (br q, BH_3_, monomer), −2.61 (br d, BH_3_, dimer, chelating), −1.74 (br q, BH_3_, dimer, bridging), 1.00 (s, CH(C*H*_3_)_2_, dimer), 1.23 (s, CH(C*H*_3_)_2_, monomer), 1.66 (s, C*H*(CH_3_)_2_, dimer), 1.95 (s, C*H*(CH_3_)_2_, monomer). ^11^B NMR (128 MHz, C_6_D_6_, δ): −33.9 (br s, fwhm = 320 Hz, monomer), −31.0 (br s, dimer). IR (KBr) ν̅_max_ (cm^–1^): 2969 (vs), 2960 (vs), 2931 (s), 2813 (w), 2763 (w), 2732 (w), 2723 (w), 2431 (vs), 2398 (w), 2359 (s), 2247 (br), 2228 (vs), 1463 (s), 1386 (s), 1366 (m), 1292 (w), 1240 (vs), 1185 (m), 1159 (m), 1101 (w), 1078 (w), 1060 (vs), 1036 (m), 1026 (m), 966 (w), 928 (m), 885 (s), 797 (m), 771 (m), 727 (s), 711 (m), 680 (vs), 653 (m), 638 (m), 629 (w).

### Tris(diisopropylphosphinodiboranato)terbium(III), Tb(H_3_BP^*i*^Pr_2_BH_3_)_3_ (Tb-^*i*^Pr)

TbI_3_ (0.100 g, 0.185 mmol) and K(H_3_BP^*i*^Pr_2_BH_3_) (0.103 g, 0.560 mmol) were loaded into a 5 mL FTS ball-milling jar with two 5 mm stainless steel balls and approximately 15 drops of pentane. The jar was hermetically sealed using electrical tape, transferred to an FTS shaker mill, and milled for 90 min at 1800 rpm. The jar was transferred to a glovebox and opened to reveal a gray paste. The contents were suspended in Et_2_O (15 mL), stirred for 15 min, and filtered through a plug of Celite. Pentane (20 mL) was added to the clear solution to precipitate the unreacted ligand salt, and the mixture was stirred for 45 min. The mixture was filtered, the filtrate was evaporated to dryness under a vacuum, and the residue was dissolved in DCM (5 mL). Vapor diffusion with pentane at −30 °C yielded small clear blocks after five months when the laboratory was reopened after the COVID-19 shutdown. Yield: 15.2 mg (14%). ^1^H NMR (400 MHz, C_6_D_6_, δ): −356.9 (br s, fwhm = 7000 Hz, BH_3_), −2.03 (br s, fwhm = 110 Hz, C*H*(CH_3_)_2_, monomer), 1.13 (br s, fwhm = 110 Hz, CH(C*H*_3_)_2_, monomer), 14.33 (br s, dimer), 15.18 (br s, dimer). ^11^B NMR (128 MHz, C_6_D_6_, δ): −741.6 (br s, dimer, bridging), −546.5 (br s, fwhm = 520 Hz, monomer), and −502.2 (br s, dimer, chelating). IR (KBr) ν̅_max_ (cm^–1^): 2968 (vs), 2931 (s), 2896 (s), 2871 (s), 2813 (w), 2763 (w), 2732 (w), 2723 (sh), 2434 (s), 2404 (w), 2361 (m), 2341 (w), 2258 (sh), 2229 (vs), 1462 (vs), 1386 (m), 1365 (m), 1245 (vs), 1158 (m), 1143 (sh), 1102 (w), 1060 (vs), 1037 (m), 1025 (m), 966 (w), 927 (m), 885 (s), 798 (m), 772 (m), 728 (m), 704 (w), 683 (vs), 651 (w), 639 (m).

### Tris(diisopropylphosphinodiboranato)erbium(III), Er(H_3_BP^*i*^Pr_2_BH_3_)_3_ (Er-^*i*^Pr)

ErI_3_ (0.100 g, 0.182 mmol) and K(H_3_BP^i^Pr_2_BH_3_) (0.103 g, 0.560 mmol) were loaded into a 5 mL FTS ball-milling jar with two 5 mm stainless steel balls and approximately 15 drops of pentane. The jar was hermetically sealed, transferred to a FlackTek SpeedMixer, and milled three times at 1800 rpm for 5 min for each cycle. The jar was transferred to a glovebox and opened to reveal a gray paste. The contents were suspended in Et_2_O (15 mL), stirred for 15 min, and filtered through a plug of Celite. Pentane (20 mL) was added to the light pink solution with stirring, which yielded a precipitate presumed to be an unreacted ligand salt. The mixture was filtered through a plug of Celite, and the filtrate was evaporated to dryness under vacuum. The residue was dissolved in DCM (5 mL). Vapor diffusion with pentane at −30 °C yielded small pink blocks that were collected after one month. Yield: 41.1 mg (38%). ^1^H NMR (400 MHz, C_6_D_6_, δ): −182.0 (br s, fwhm = 9000 Hz, BH_3_, monomer), −4.75 (br s, CH(C*H*_3_)_2_, dimer, bridging), 3.42 (br s, fwhm = 75 Hz, CH(C*H*_3_)_2_, monomer), 10.68 (br s, CH(C*H*_3_)_2_, dimer, chelating). ^11^B NMR (128 MHz, C_6_D_6_, δ): −427.7 (br s, dimer, bridging), −270.5 (br s, fwhm = 320 Hz, monomer), −234.3 (br s, dimer, chelating). IR (KBr) ν̅_max_ (cm^–1^): 2967 (vs), 2961 (vs), 2931 (s), 2896 (m), 2872 (s), 2849 (w), 2812 (w), 2763 (w), 2733 (w), 2721 (w), 2438 (vs), 2420 (w), 2409 (s), 2368 (m), 2346 (w), 2251 (sh), 2232 (vs), 1463 (s), 1451 (sh), 1386 (m), 1365 (m), 1262 (m), 1247 (vs), 1159 (m), 1135 (w), 1102 (w), 1062 (vs), 1053 (sh), 1038 (s), 1026 (m), 967 (w), 928 (m), 885 (m), 800 (m), 729 (m), 705 (w), 686 (s), 649 (m), 642 (m).

### Tris(dimethylphosphinodiboranato)uranium(III), U(H_3_BPMe_2_BH_3_)_3_ (U-Me)

UI_3_(1,4-dioxane)_1.5_ (0.105 g, 0.140 mmol) and K(H_3_BPMe_2_BH_3_) (0.0569 g, 0.452 mmol) were stirred in Et_2_O overnight. The reaction mixture was evaporated to dryness under a vacuum and extracted with Et_2_O. The mixture was filtered and layered with pentane to yield a few small, dark red prisms that were suitable for XRD analysis. This chemistry was investigated prior to our discovery that mechanochemical methods are more effective for preparing PDB complexes in higher yields for analysis. This reaction was not revisited for further optimization and more thorough characterization, but we wish to report the structure of U-Me here for comparison given its parallels to the structure of Nd-Et.

### Crystallographic Studies

Single-crystal X-ray diffraction data were collected as previously described.^14,16,20,21^ All crystallographic data except those for Tb-^*i*^Pr were collected on a Bruker Nonius Kappa ApexII instrument equipped with a charge-coupled-device (CCD) detector. The data for Tb(H_3_BP^*i*^Pr_2_BH_3_)_3_ were collected on a Bruker D8 Venture Duo instrument equipped with a Bruker photon III detector. Both instruments were equipped with graphite monochromatized Mo Kα radiation (λ = 0.71073 Å). Samples of Nd-^*i*^Pr and U-Me were cooled to 180 and 190 K, respectively, using an Oxford Cryostream 700 low temperature device. All other samples were cooled to 150 K. Data were collected using phi and omega scans and corrected for absorption using redundant reflections and the SADABS^41^ program. Structures were solved with intrinsic phasing (SHELXT)^42^ and subsequent least-squares refinement (SHELXL),^43^ which confirmed the positions of all non-hydrogen atoms. All hydrogen atom positions were idealized and allowed to ride on the attached carbon and boron atoms. B–H distances were fixed at 1.20 Å. Structure solution and refinement were performed with Olex2.^44^ Publication figures were made using Mercury version 4.3.1 or Olex2.^44,45^ Crystallographic data and refinement details for each structure are provided in Table S1 in the Supporting Information.

### Computational Details

Prior to DFT geometry optimizations, a low-level DFT-tight binding (GFN2-xTB) conformational search was performed for monomer and dimer structures of Nd-Et, Nd-^*i*^Pr, Nd-Ph, and Nd-^*t*^Bu complexes using the Conformer-Rotamer Ensemble Sampling Tool (CREST) in the xtb program.^30^ The lowest energy conformer for the monomer was taken as the starting structure for subsequent optimization with DFT. In the case of the dimers, starting geometries were taken both from available X-ray diffraction structures and from the lowest conformer predicted by CREST. For each dimer, the two structures were optimized, and that with the lowest energy was used in subsequent analysis. DFT geometry optimizations were performed, followed by harmonic vibrational analysis. All structures were confirmed as minima (with few exceptions, *vide infra*), and free energies are reported using the standard harmonic oscillator, rigid rotor approximations. The TPSS functional with Grimme’s D3 correction was employed (TPSS-D3) with the original damping function. The resolution of identity (RI) approximation was used for integral evaluation.^46−50^ The def2-TZVP basis is used on all atoms with the exception of uranium where the def-TZVP basis was used for uranium and for carbon where the def2-SV(P) basis was used.^51−58^ The smaller basis set is used on carbon to reduce the computational cost for the largest dimers. For the model oligomers of Nd-Et and Nd-^*i*^Pr, molecular geometries were optimized with def2-TZVP on Nd and def2-SV(P) for all other atoms using same functional. Once more, this was due to system size and the resulting computational cost. The SCF energy was converged to 10^–7^ a.u., and the Cartesian gradient was converged to 10^–4^ a.u. Single point calculations including the conductor-like screening model (COSMO)^32^ were performed on the gas phase geometries to account for solvation using a dielectric constant of 2.274 for benzene. All DFT calculations were performed as implemented in the Turbomole program package. Some of the dimers have very small (<15 cm^–1^) imaginary modes associated with methyl rotations. These do not impact the computed free energies since the quasiharmonic correction suggested by Cramer and Truhlar in which all normal modes less than 100 cm^–1^ are replaced with 100 cm^–1^ is employed.^59^ Specifically, free energies were corrected using the single point energies in benzene, computed at 298.15 K, and assumed a concentration of 1 M for all reactants and products.

## Data Availability

A data set collection of computational results is available in the ioChem-BD repository^60^ and can be accessed online using the following link (https://iochem-bd.bsc.es/browse/review-collection/100/305319/68e20fc7fd8dc58691e7d63f). The input and output files are also available in the FigShare repository (https://figshare.com/s/a98c4ccb7d38476600c0)

## Supporting Information Available

Optimized structures (ZIP)
Crystallographic details, NMR and IR spectra, tabulated DFT data and structures (PDF)
