# Supplementary material for: The Influence of Phosphorus Substituents on the Structures and Solution Speciation of Trivalent Uranium and Lanthanide Phosphinodiboranates
Source: Inorg Chem. 2023 Nov 27;63(21):9451–63. doi: 10.1021/acs.inorgchem.3c02773 (PMC11134491; doi:10.1021/acs.inorgchem.3c02773)
Supplement: Supplementary file 2 — ic3c02773_si_002.pdf [file ic3c02773_si_002.pdf]

SUPPORTING INFORMATION

The Influence of Phosphorus Substituents on the Structures and Solution Speciation of Trivalent Uranium and Lanthanide Phosphinodiboranates

Joshua C. Zgrabik,[1] Rina Bhowmick,[2] Francesca D. Eckstrom,[1] A. Rayford Harrison,[1] Taylor V. Fetrow,[1] Anastasia V. Blake,[1] Bess Vlaisavljevich,[2,*] Scott R. Daly[1,*]

[1]Department of Chemistry, The University of Iowa, E331 Chemistry Building, Iowa City, Iowa 52242, United States

[2]Department of Chemistry, The University of South Dakota, 414 E Clark St., Vermillion SD, 57069, United States.

Corresponding email: scott-daly@uiowa.edu; Bess.Vlaisavljevich@usd.edu

Table of Contents

I. XRD data S2
II. NMR spectra S6
III. IR spectra S16
IV. DFT data S18

I. XRD data

Table S1. Crystallographic data.

	**U-^{i}Pr**	**Nd-^{i}Pr**	**Sm-^{i}Pr**	**Tb-^{i}Pr**	**Er-^{i}Pr**	**Nd-Et**	**U-Me**	**Nd-Et-THF**
Identifier	Dal20_29	Dal19_60	Dal18_45	Dal22_2	Dal18_52	Dal20_61	Dal17_35	Dal20_58
Formula	$C_{18}H_{60}B_6P_3U$	$C_{18}H_{60}B_6P_3Nd$	$C_{18}H_{60}B_6P_3Sm$	$C_{18}H_{60}B_6P_3Tb$	$C_{18}H_{60}B_6P_3Er$	$C_{12}H_{48}B_6P_3Nd$	$C_6H_{36}B_6P_3U$	$C_{24}H_{68}B_6O_3P_3Nd$
FW (g·mol^{-1})	672.46	578.67	584.78	593.35	601.69	494.51	504.15	706.79
crystal system	monoclinic	monoclinic	monoclinic	monoclinic	monoclinic	monoclinic	monoclinic	triclinic
space group	$P2_1/c$	$P2_1/c$	$P2_1/c$	$P2_1/c$	$P2_1/c$	C2/c	C2/c	P-1
a (Å)	10.9408(13)	10.9622(11)	10.9579(11)	10.9639(11)	10.9819(11)	20.306(4)	18.0385(9)	10.5933(14)
b (Å)	13.1542(15)	13.0969(13)	13.1056(13)	13.1097(13)	12.9649(13)	10.264(2)	9.6543(5)	13.6785(19)
c (Å)	23.150(3)	23.014(2)	22.776(2)	22.694(2)	22.500(2)	13.073(3)	12.6841(7)	15.145(2)
α (deg)	90	90	90	90	90	90	90	78.963(5)
β (deg)	95.640(5)	95.149(5)	94.672(5)	94.521(5)	94.345(5)	110.268(5)	106.534(4)	70.463(5)
γ (deg)	90	90	90	90	90	90	90	73.164(5)
volume (Å)^3	3315.6(7)	3290.8(5)	3260.0(5)	3251.7(5)	3194.3(5)	2556.0(9)	2117.6	1968.6(5)
Z	4	4	4	4	4	4	4	2
ρ_{calc} (g cm^{-3})	1.347	1.168	1.191	1.212	1.251	1.285	1.581	1.192
μ (mm^{-1})	5.045	1.728	1.953	2.327	2.782	2.212	7.870	1.461
F (000)	1340	1212	1220	1232	1244	1020	956	742
θ range (deg)	25.242	25.242	25.242	25.492	25.242	25.242	25.242	25.242
R (int)	0.0499	0.0312	0.0269	0.0172	0.0466	0.0792	0.0649	0.0508
data/restraints/ parameters	6743/0/271	7257/0/271	7102/0/271	7741/0/337	7824/0/270	2621/31/127	2159/0/80	8057/42/356
GOF	1.031	1.070	1.040	1.075	1.042	1.013	1.032	1.094
R_1 [$I > 2\sigma(I)$]^a	0.0263	0.0232	0.0186	0.0147	0.0299	0.0387	0.0335	0.0668
wR_2 (all data)^b	0.0543	0.0486	0.0420	0.0328	0.0647	0.0897	0.0672	0.1774
largest peak/hole (e · Å^{-3})	0.750/-0.470	0.700/-0.304	0.429/-0.244	0.419/-0.280	0.754/-0.443	0.774/-0.853	0.749/-1.137	1.617/-1.784
temp (K)	150(2)	180(2)	150(2)	150(2)	150(2)	150(2)	190(2)	150(2)

$^a R_1 = \Sigma\ |F_o| - |F_c|\ |\ /\ |\ |\Sigma|F_o|$ for reflections with $F_o^2 > 2\sigma(F_o^2)$.

$^b wR_2 = [\Sigma w(F_o^2 - F_c^2)^2\ /\ \Sigma(F_o^2)^2]^{1/2}$ for all reflections.

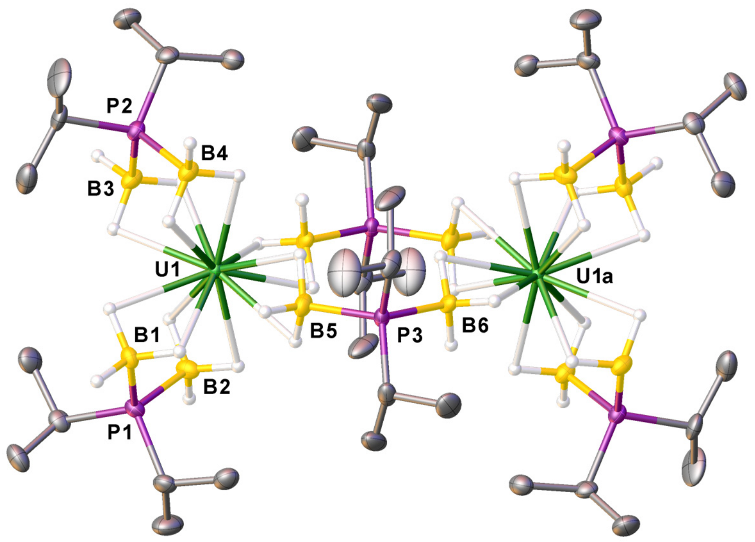

Figure S1. Molecular structure of U($H_3BP^iPr_2BH_3$)$_3$ (**U-*i*Pr**) with thermal ellipsoids at 35% probability. Hydrogen atoms attached to carbon were omitted from the figure.

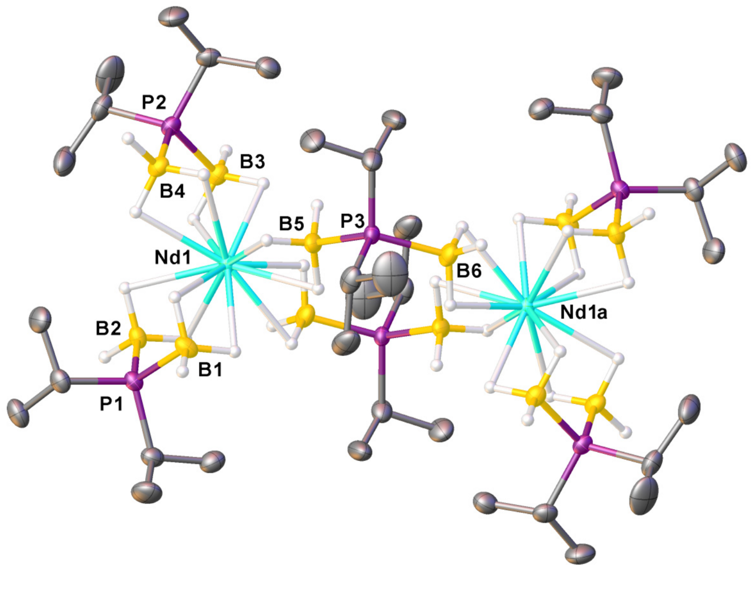

Figure S2. Molecular structure of Nd($H_3BP^iPr_2BH_3$)$_3$ (**Nd-*i*Pr**) with thermal ellipsoids at 35% probability. Hydrogen atoms attached to carbon were omitted from the figure.

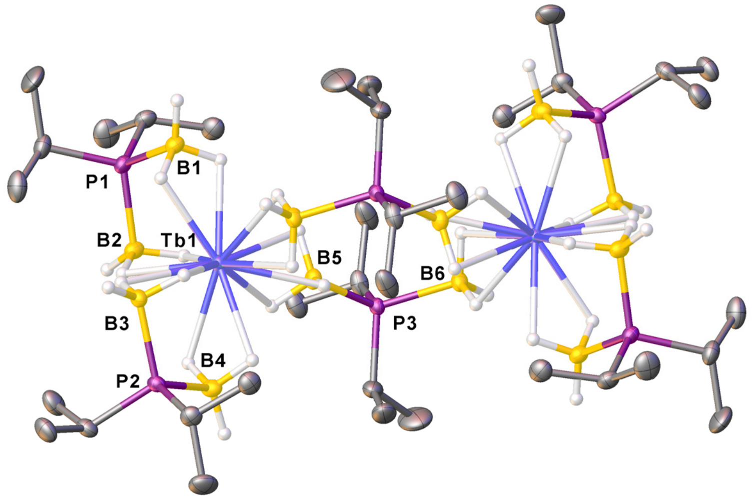

Figure S3. Molecular structure of $Tb(H_3BP^iPr_2BH_3)_3$ (**Tb-^{i}Pr**) with thermal ellipsoids at 35% probability. Hydrogen atoms attached to carbon were omitted from the figure.

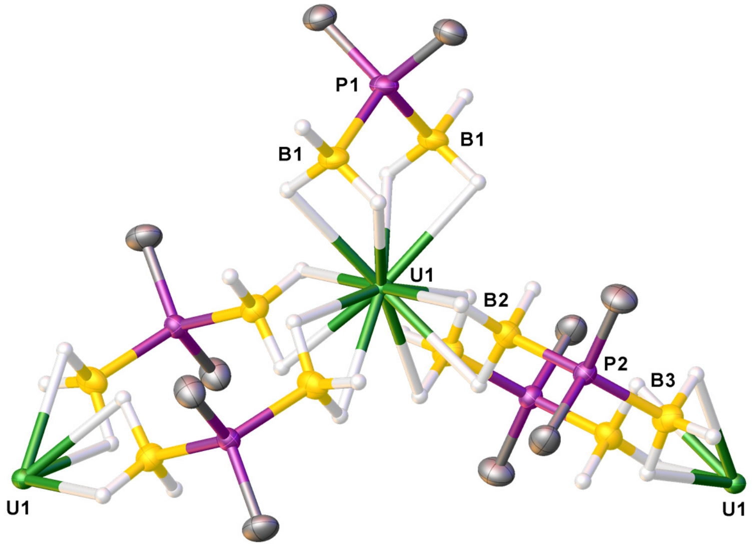

Figure S4. Molecular structure of $U(H_3BPMe_2BH_3)_3$ (**U-Me**) with thermal ellipsoids at 35% probability. Hydrogen atoms attached to carbon were omitted from the figure.

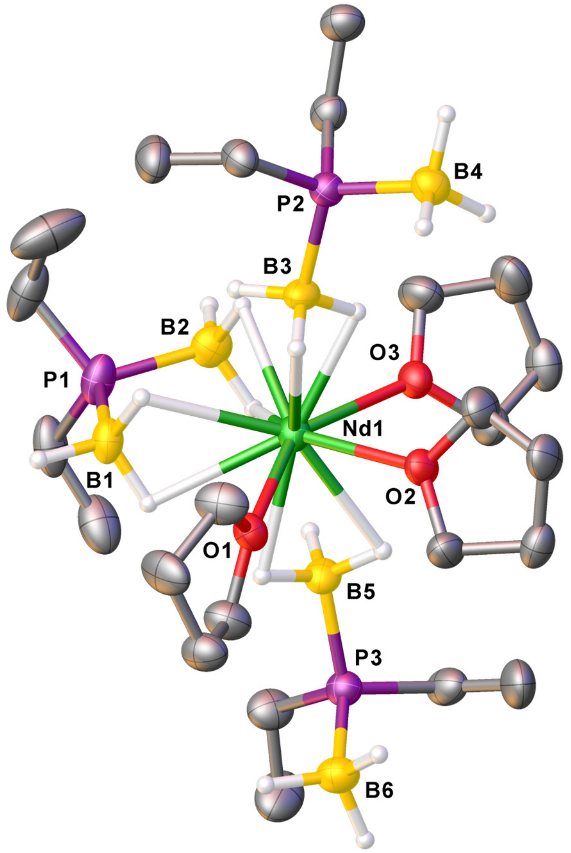

Figure S5. Molecular structure of Nd($H_3BPEt_2BH_3$)$_3$(THF)$_3$ (**Nd-Et-THF**) with thermal ellipsoids at 35% probability. Hydrogen atoms attached to carbon and a disordered carbon on THF were omitted from the figure.

II. NMR spectra

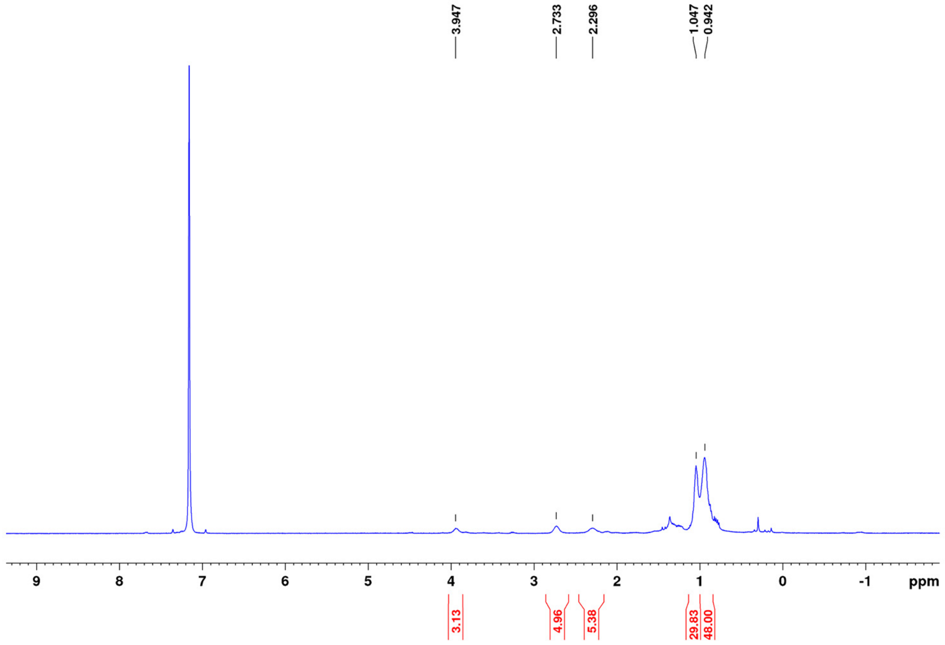

Figure S6. ^{1}H NMR spectrum of $U(H_3BP^{i}Pr_2BH_3)_3$ in C_6D_6 (isopropyl region).

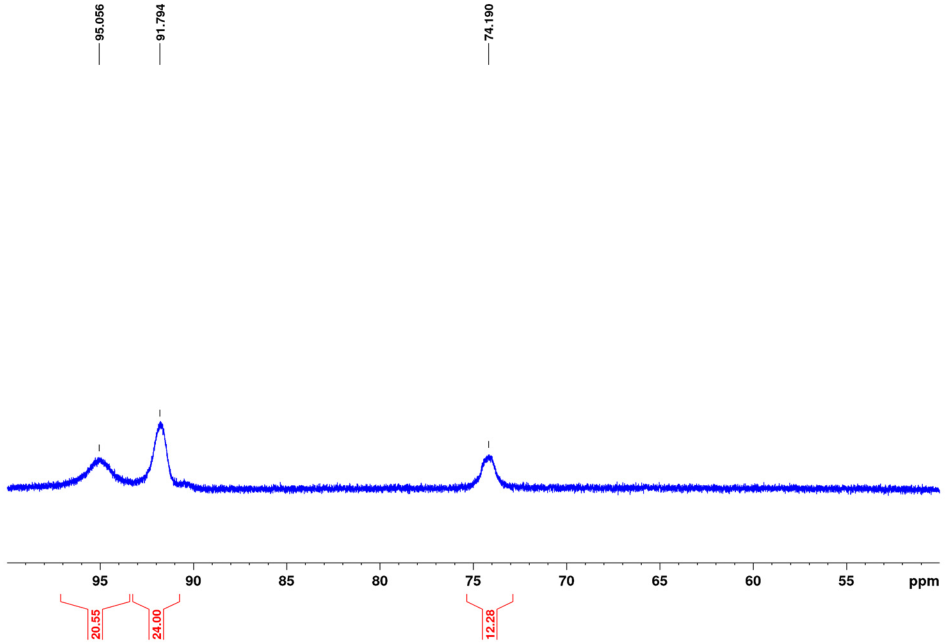

Figure S7. ^{1}H NMR spectrum of $U(H_3BP^{i}Pr_2BH_3)_3$ in C_6D_6 (BH_3 region)

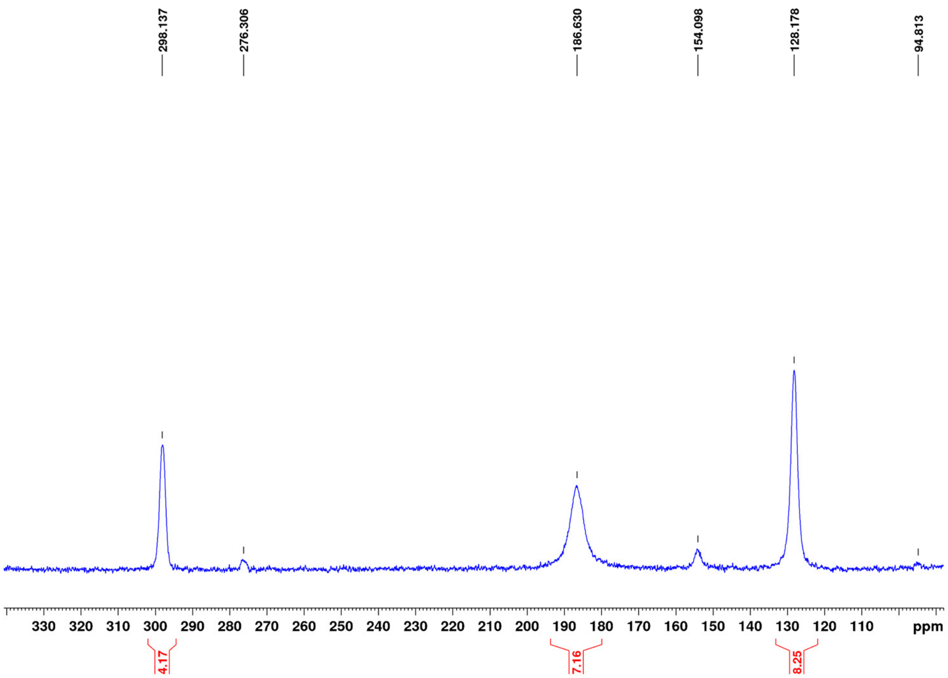

Figure S8. ^{11}B NMR spectrum of $U(H_3BP^{i}Pr_2BH_3)_3$ in C_6D_6.

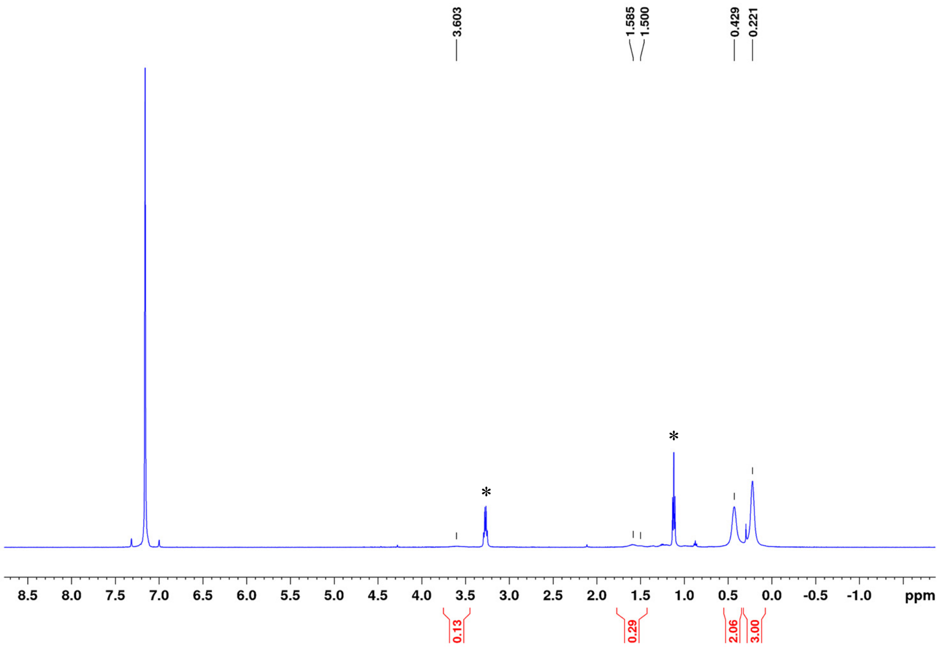

Figure S9. ^{1}H NMR spectrum of $U(H_3BPEt_2BH_3)_3$ in C_6D_6 (ethyl region). The * indicates resonances associated with residual Et_2O.

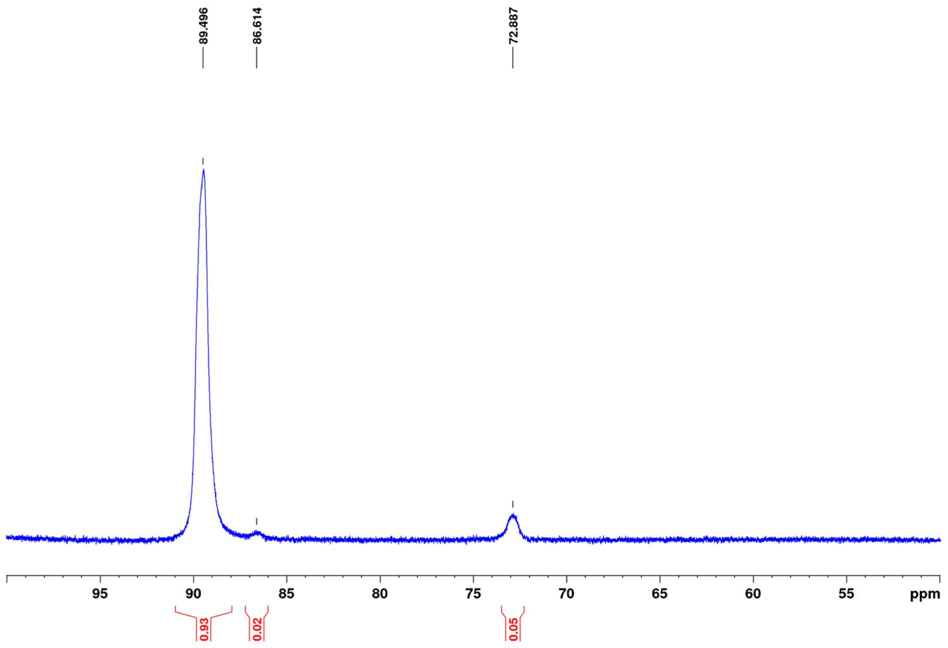

Figure S10. ^{1}H NMR spectrum of $U(H_3BPEt_2BH_3)_3$ in C_6D_6 (BH_3 region).

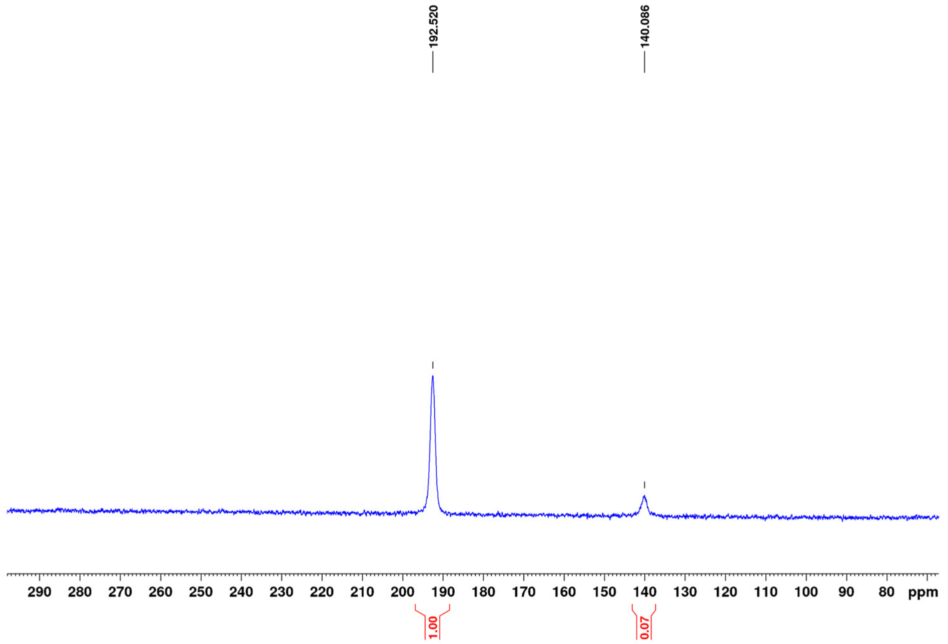

Figure S11. ^{11}B NMR spectrum of $U(H_3BPEt_2BH_3)_3$ in C_6D_6.

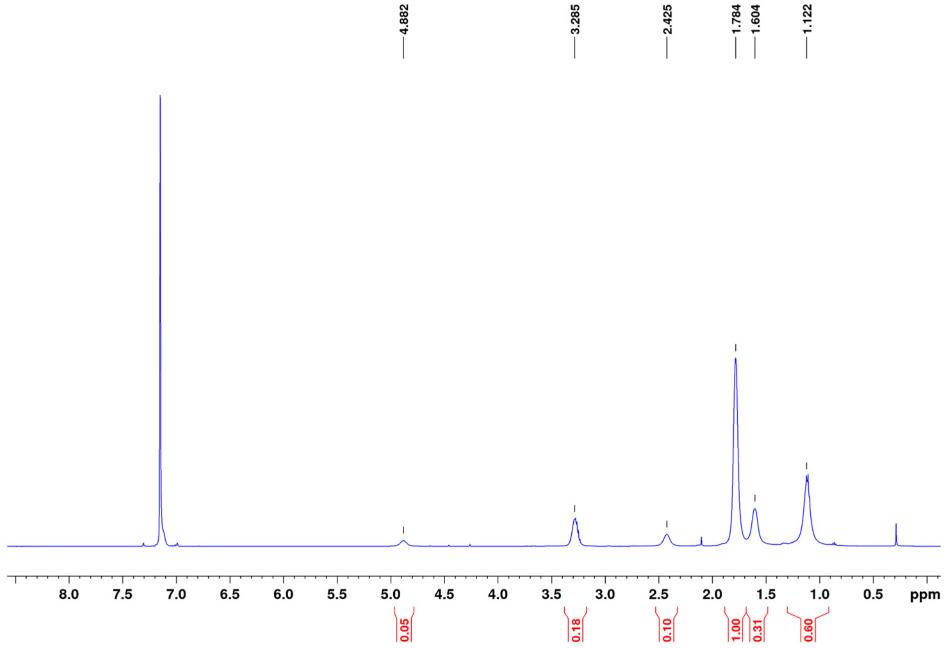

Figure S12. ^{1}H NMR spectrum of $Nd(H_3BP^iPr_2BH_3)_3$ in C_6D_6 (isopropyl region).

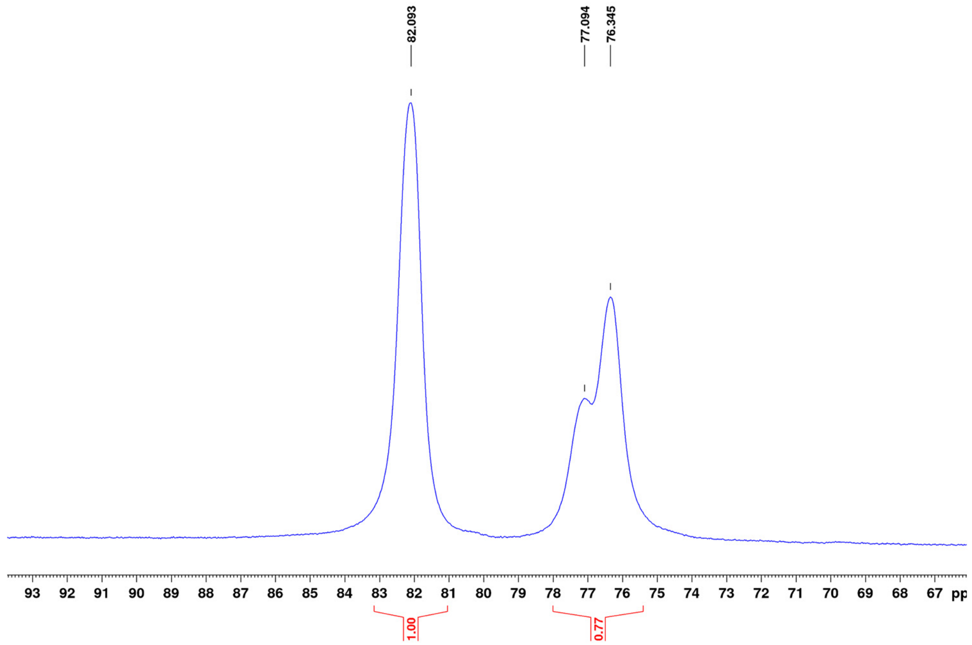

Figure S12. ^{1}H NMR spectrum of $Nd(H_3BP^iPr_2BH_3)_3$ in C_6D_6 (BH_3 region).

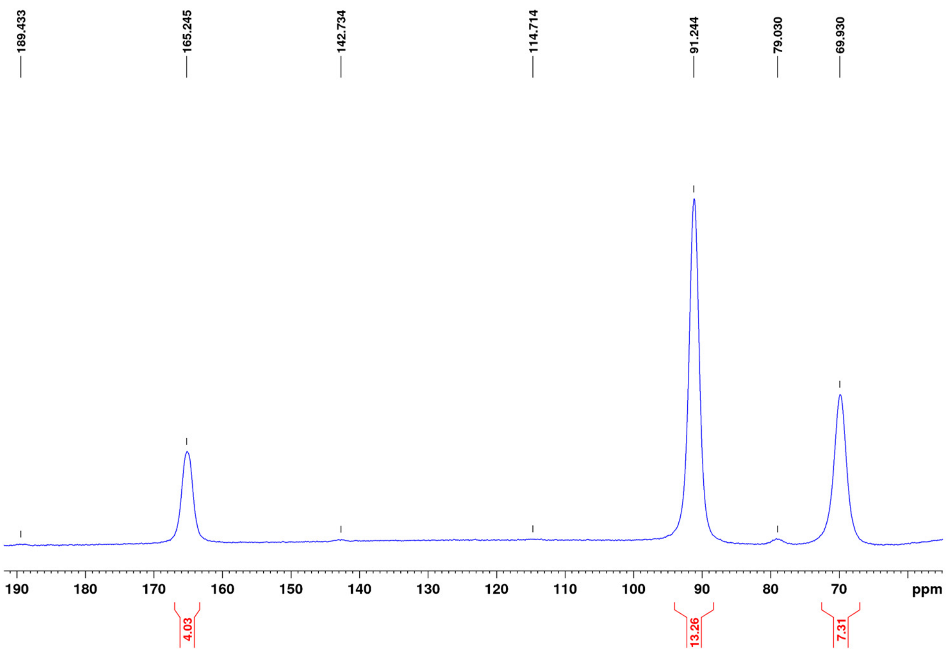

Figure S13. ^{11}B NMR spectrum of $Nd(H_3BP^iPr_2BH_3)_3$ in C_6D_6.

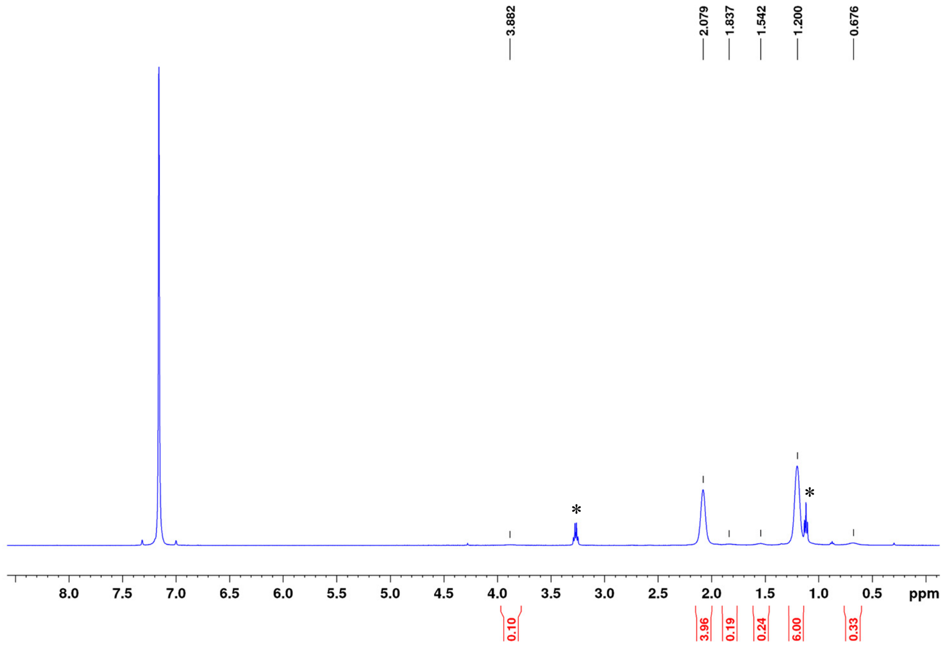

Figure S14. ^{1}H NMR spectrum of $Nd(H_3BPEt_2BH_3)_3$ in C_6D_6 (ethyl region). The * indicates resonances associated with residual Et_2O.

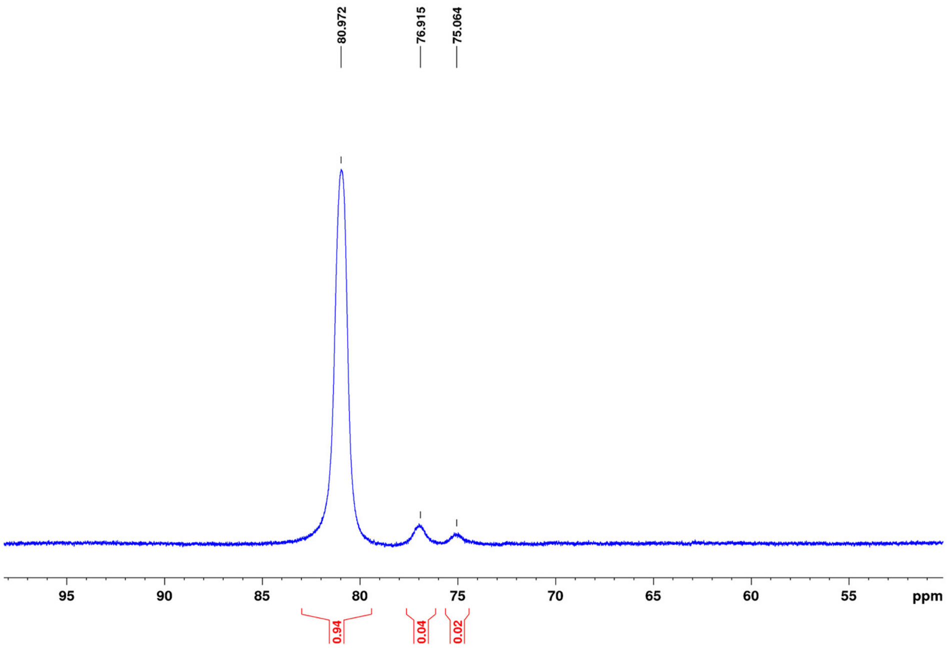

Figure S15. ^{1}H NMR spectrum of $Nd(H_3BPEt_2BH_3)_3$ in C_6D_6 (BH_3 region).

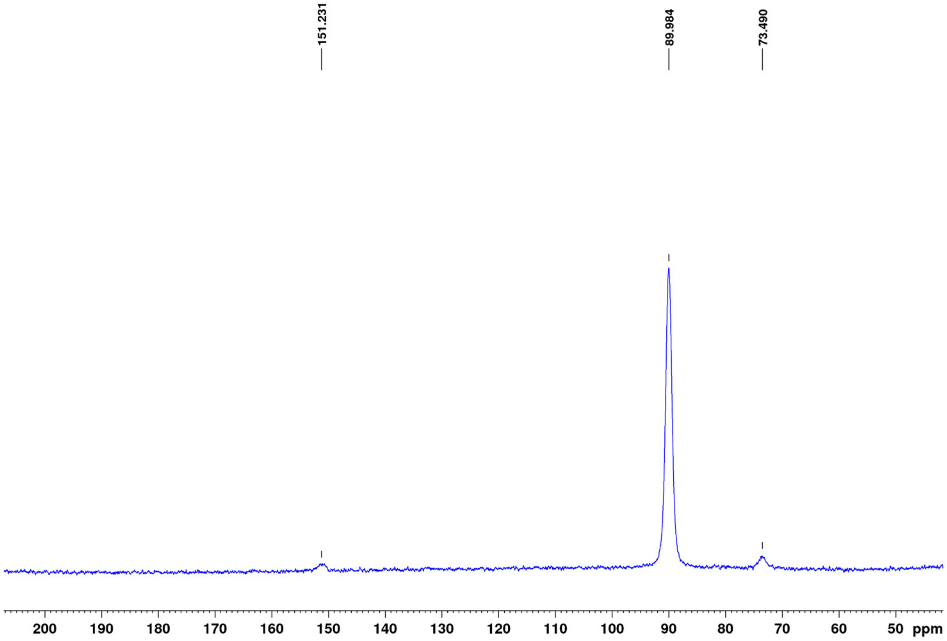

Figure S16. ^{11}B NMR spectrum of $Nd(H_3BPEt_2BH_3)_3$ in C_6D_6.

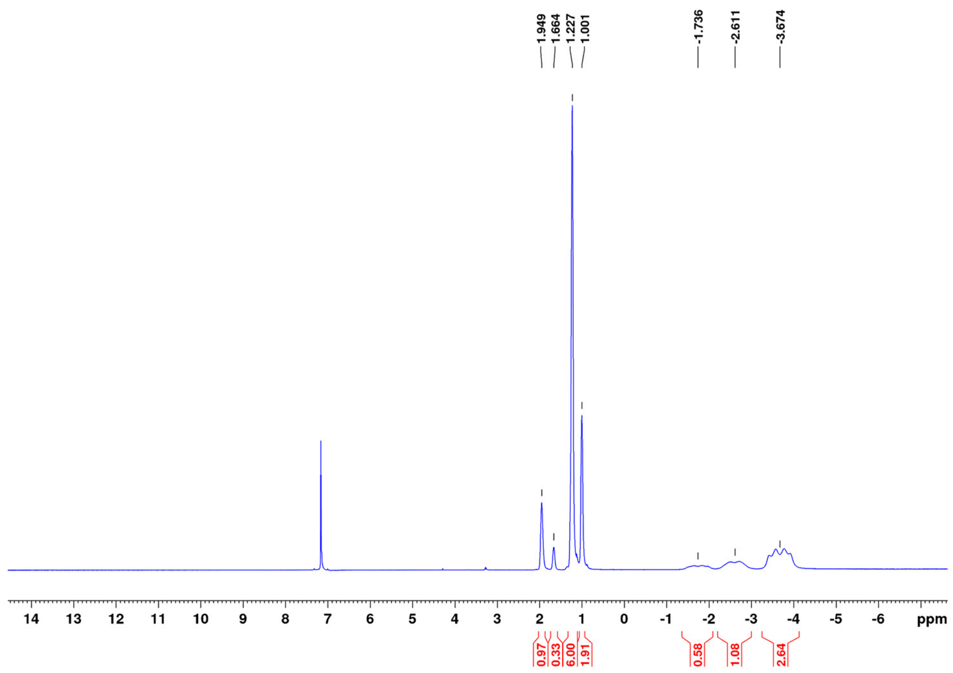

Figure S17. ^{1}H NMR spectrum of $Sm(H_3BP^{i}Pr_2BH_3)_3$ in C_6D_6.

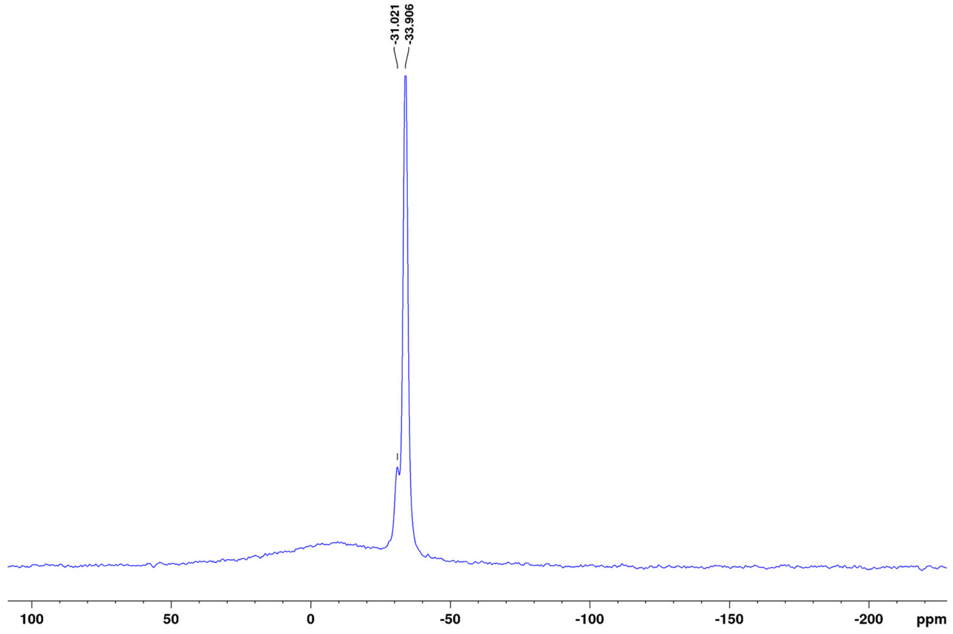

Figure S18. ^{11}B NMR spectrum of $Sm(H_3BP^{i}Pr_2BH_3)_3$ in C_6D_6.

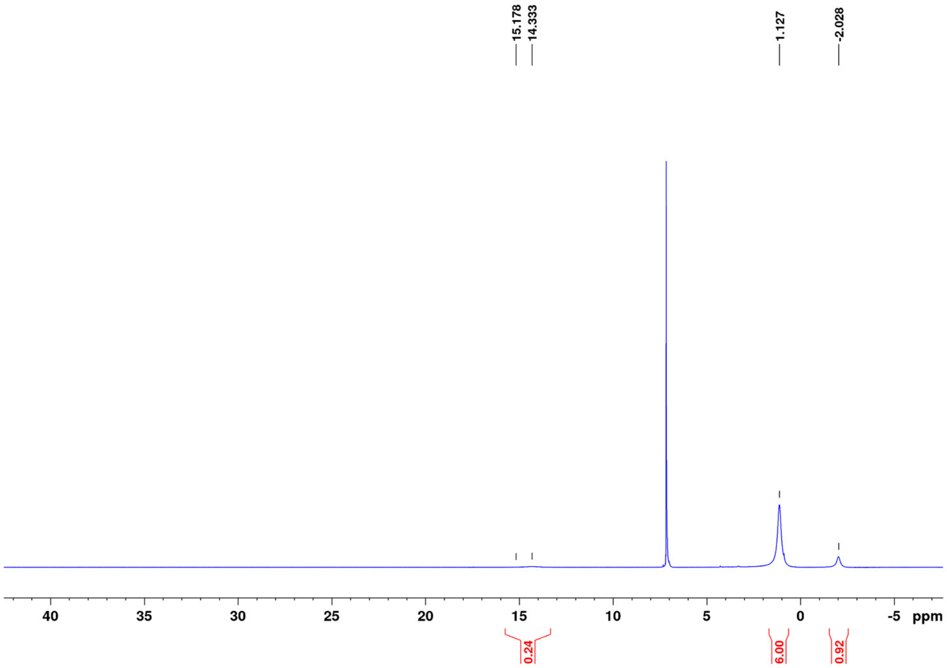

Figure S19. ^{1}H NMR spectrum of $Tb(H_3BP^{i}Pr_2BH_3)_3$ in C_6D_6 (isopropyl region).

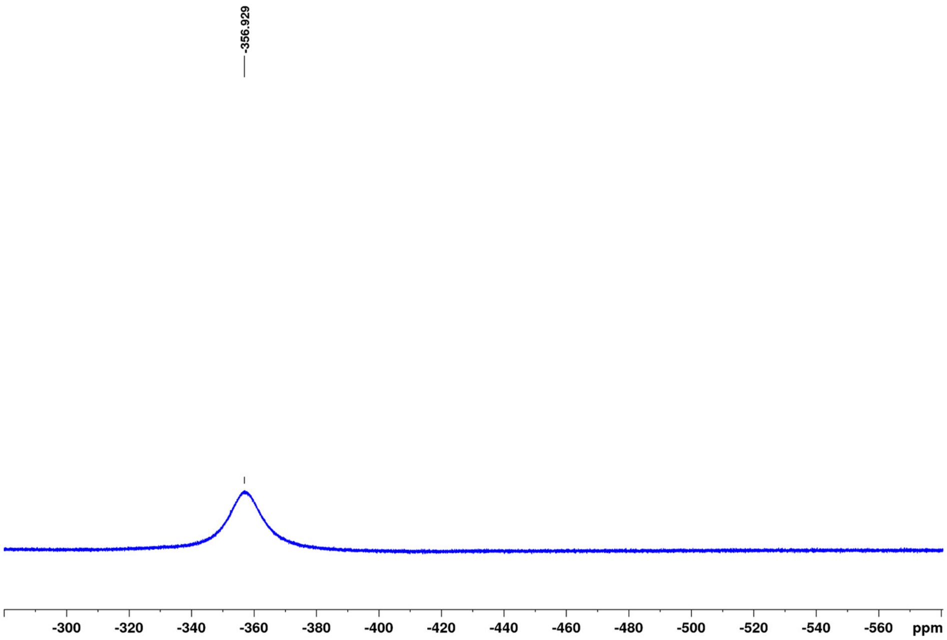

Figure S20. ^{1}H NMR spectrum of $Tb(H_3BP^{i}Pr_2BH_3)_3$ in C_6D_6 (BH_3 region).

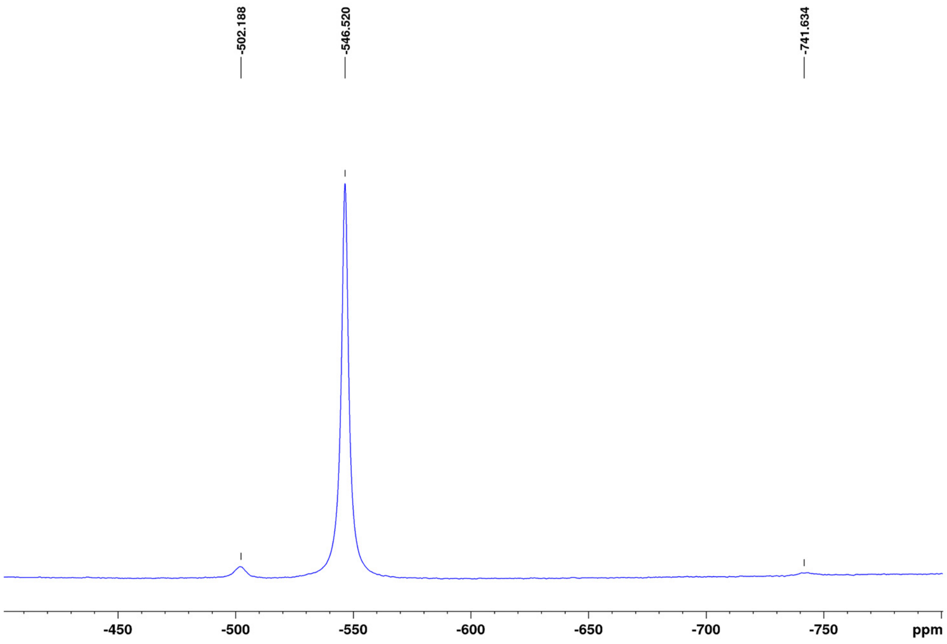

Figure S21. ^{11}B NMR spectrum of $Tb(H_3BP^{i}Pr_2BH_3)_3$ in C_6D_6.

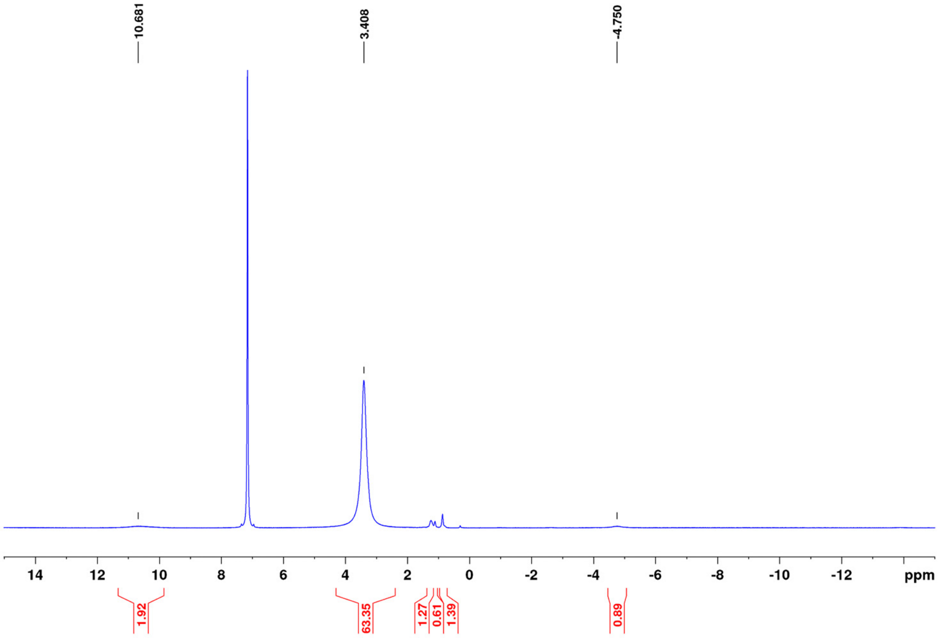

Figure S22. ^{1}H NMR spectrum of $Er(H_3BP^{i}Pr_2BH_3)_3$ in C_6D_6 (isopropyl region).

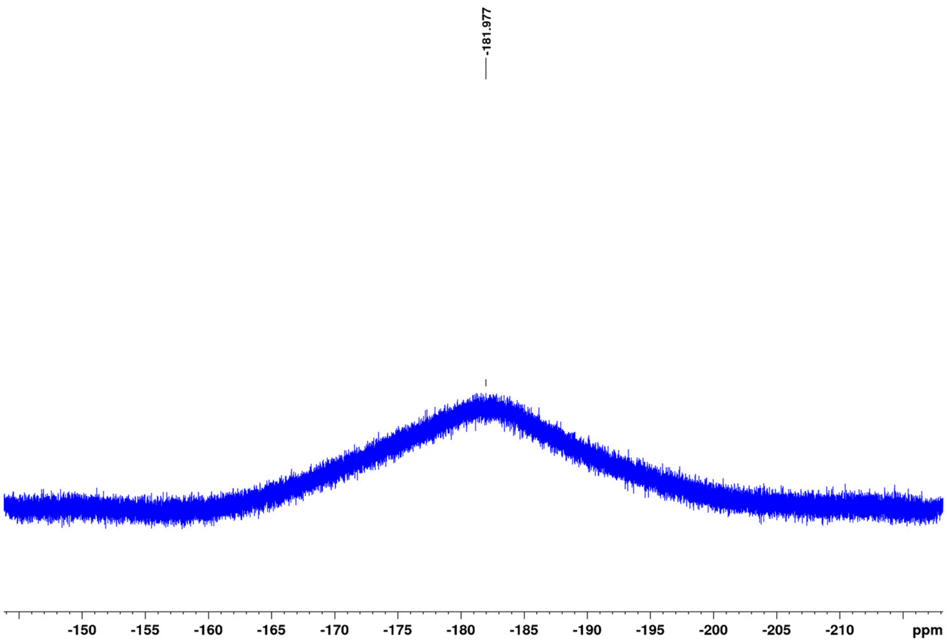

Figure S23. ^{1}H NMR spectrum of $Er(H_3BP^iPr_2BH_3)_3$ in C_6D_6 (BH_3 region).

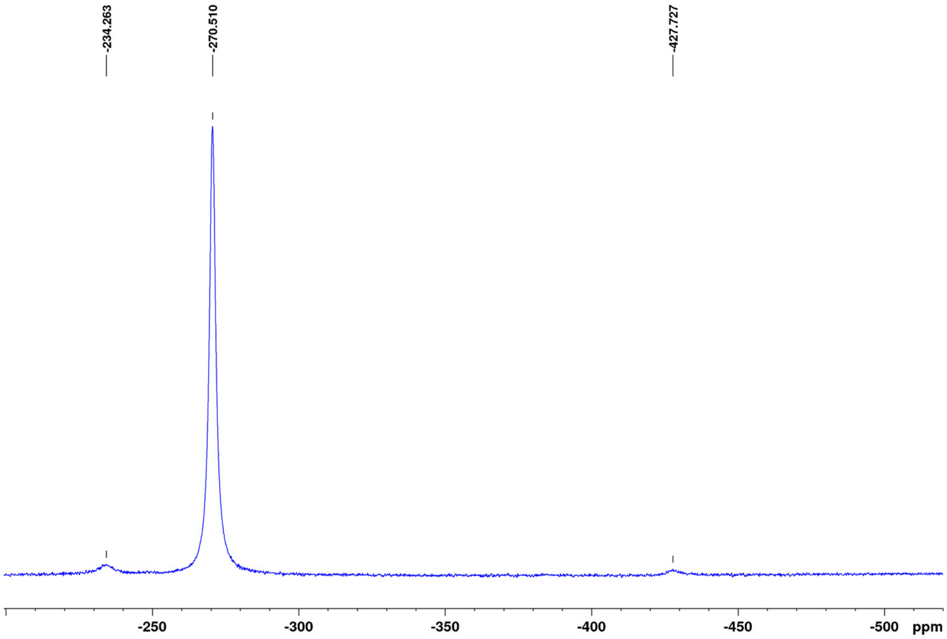

Figure S24. ^{11}B NMR spectrum of $Er(H_3BP^iPr_2BH_3)_3$ in C_6D_6.

III. IR spectra

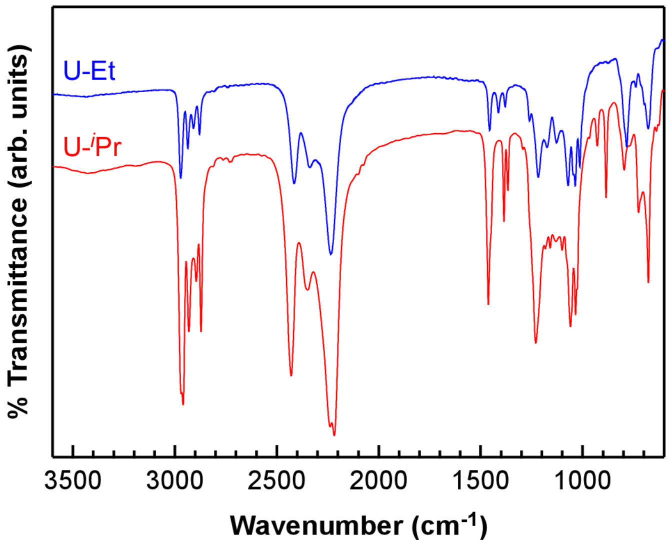

Figure S25. KBr IR spectra of $U(H_3BP^{i}Pr_2BH_3)_3$ (red) and $U(H_3BPEt_2BH_3)_3$ (blue).

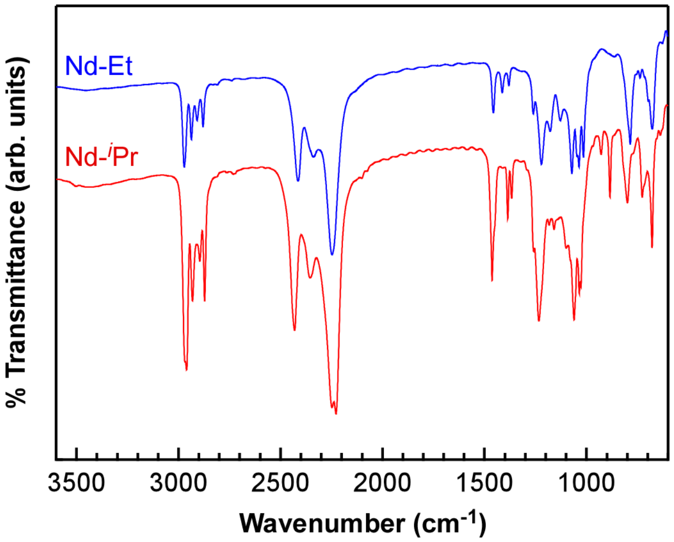

Figure S26. KBr IR spectra of $Nd(H_3BP^{i}Pr_2BH_3)_3$ (red) and $Nd(H_3BPEt_2BH_3)_3$ (blue).

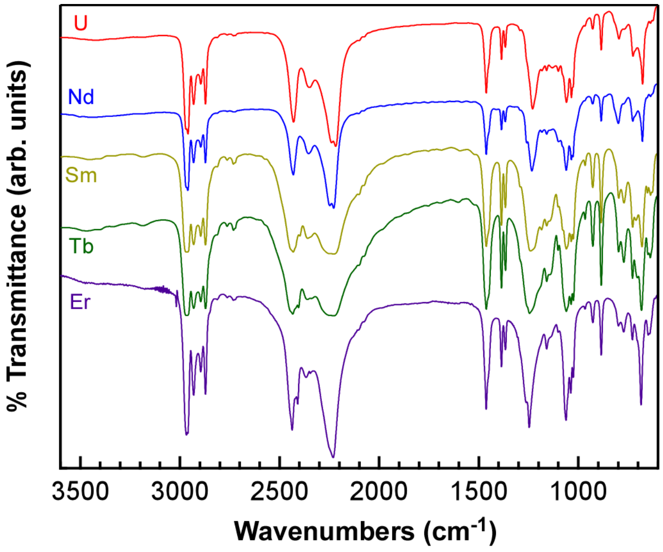

Figure S27. KBr IR spectra of $M(H_3BP^iPr_2BH_3)_3$ complexes (M = U, Nd, Sm, Tb, and Er).

IV. DFT data

Table S2. Selected bond distances (Å) and ligand B-P-B angles (°) of dimers from the RI-TPSS-D3/def2-TZVP (def-TZVP for U, def2-SV(P) for C) on optimized structures of the dimers.

Complex	M-B (Å) Chelating	M-B (Å) Bridging		Chelating Angle	Bridging Angle	Δ Angle
**Nd-Et (model)**	2.793	2.672	2.874	110.7	119.2	8.5
**Nd-*i*Pr**	2.801	2.630	2.865	110.1	118.7	8.6
**Nd-Ph**	2.796	2.600	2.826	111.0	117.7	6.8
**Nd-*t*Bu**	2.820	2.607	2.800	107.1	117.4	10.3
**U-Et (model)**	2.787	2.685	2.900	110.9	118.6	7.6
**U-*i*Pr**	2.839	2.584	2.851	106.4	117.8	11.4
**U-Ph**	2.807	2.574	2.837	109.9	118.1	8.2
**U-*t*Bu**	2.846	2.599	2.754	104.9	113.6	8.7
**Sm-*i*Pr**	2.778	2.681	2.865	110.5	116.9	6.4
**Tb-*i*Pr**	2.773	2.593	2.788	108.3	121.5	13.1
**Er-*i*Pr**	2.744	2.531	2.766	103.5	118.1	14.6

Table S3. Average M-B bond distances (Å) and B-P-B angles (°) from the XRD structures of the dimers.

Complex	M-B (Å) Chelating	M-B (Å) Bridging (short)	M-B (Å) Bridging (long)	B-P-B (°) Chelating	B-P-B (°) Bridging
**Nd-*i*Pr**	2.872	2.656	2.780	107.8	116.7
**Nd-Ph**	2.900	2.666	2.696	106.1	112.3
**Nd-*t*Bu**	2.886	2.666	2.674	105.1	108.4
**U-*i*Pr**	2.909	2.691	2.771	108.3	114.6
**U-Ph**	2.925	2.683	2.700	105.9	111.9
**U-*t*Bu**	2.920	2.690	2.694	106.6	108.3
**Sm-*i*Pr**	2.844	2.625	2.813	106.9	118.7
**Tb-*i*Pr**	2.817	2.598	2.796	105.5	119.6
**Er-*i*Pr**	2.782	2.565	2.757	104.3	120.8

Table S4. The TPSS-D3 geometry parameters and Gibbs free energy calculation with two different basis sets. In the mixed basis set def2-SV(P) is used for light atoms while def2-TZVP is used for Nd. In all calculations, def-TZVP was used on U. A single point calculation was performed on the geometry resulting from the mixed basis set with the larger basis set. The ΔG with the single-point corrected energy is reported in parentheses.

	**Nd-^{t}Bu**		**U-^{t}Bu**	
	Mixed	def2-TZVP	Mixed	def2-TZVP
M-B (chelating)	2.820	2.822	2.846	2.862
M-B (bridging)	2.703	2.696	2.677	2.617
B-P-B (chelating)	107.1	107.2	104.9	103.7
B-P-B (bridging)	117.4	116.3	113.6	108.3
ΔG	6.6 (3.9)	3.6	7.1 (6.3)	6.3

Table S5. Selected bond distances (Å) angles (°) of monomer from the RI-TPSS-D3/def2-TZVP (def-TZVP for U, def2-SV(P) for C) on optimized structures of the monomer.

Complex	M-B (Å) Chelating	B-P-B (°) chelating
**Nd-Et**	2.770	112.9
**Nd-^{i}Pr**	2.773	112.4
**Nd-Ph**	2.760	113.0
**Nd-^{t}Bu**	2.793	108.2
**U-Et**	2.766	114.8
**U-^{i}Pr**	2.794	111.0
**U-Ph**	2.752	114.6
**U-^{t}Bu**	2.813	106.8
**Sm-^{i}Pr**	2.759	112.3
**Tb-^{i}Pr**	2.741	110.2
**Er-^{i}Pr**	2.708	106.5

Table S6. The relative energy in kcal/mol structures optimized in DFT from lowest conformer from CREST and X-ray structure.

	From CREST	From XRD
**Nd-Et (model)**	0.0	Not available
**Nd-^{i}Pr**	0.0	1.8
**Nd-Ph**	2.3	0.0
**Nd-^{t}Bu**	5.9	0.0

Table S7. The thermochemical data at 298.15 K for the TPSS-D3 electronic energies, enthalpies, entropies, and free energies for reaction Dimer → 2 Monomer. Free energies have been computed assuming a concentration of 1 M for all species and benzene as the solvent.

**Complex**	**ΔE kcal·mol^{-1}**	**ΔG kcal·mol^{-1}**	**ΔH kcal·mol^{-1}**	**ΔS kcal·mol^{-1}·K^{-1}**
**U-Ph**	27.0	9.2	24.8	0.059
**Nd-Ph**	26.2	8.7	24.1	0.058
**U-^{i}Pr**	25.6	8.7	23.6	0.057
**U-^{t}Bu**	24.1	7.1	22.0	0.057
**Nd-^{t}Bu**	22.3	6.5	20.7	0.054
**U-Et**	20.5	3.8	18.5	0.056
**Nd-^{i}Pr**	19.9	3.6	18.1	0.055
**Sm-^{i}Pr**	20.4	3.5	18.3	0.056
**Nd-Et**	17.9	1.8	16.0	0.054
**Er-^{i}Pr**	18.2	1.0	15.7	0.056
**Tb-^{i}Pr**	17.4	-0.2	14.6	0.056

Details on the calculated structures of tetramers Nd-^{i}Pr-4 and Nd-Et-4. In the polymeric **Nd-Et** solid-state structure, all chelating Nd-B distances are relatively uniform. However, in the DFT optimized structures of the tetramer, the distances are slightly asymmetric (Table S8). The average Nd-B distance including all metal centers is 2.827 Å in the tetramer, while the distance is 2.933 Å in the solid. However, the two Nd centers in the middle of the tetramer are in an environment more similar to the solid than those at the end of the chain. If the average Nd-B distance is taken only for these groups, a value of 2.889 Å is obtained in better agreement with experiment. The bridging Nd-B distances result in one shorter and one longer bond distance (Table S9), consistent with experiment. The shorter Nd-B distance is 2.656 Å, a difference from experiment of -0.045 Å, and the longer Nd-B distances is 2.833, a difference from experiment of -0.037 Å. Moreover, the average chelating B-P-B angles are also in good agreement with experiment (Table S10). Specifically, the B-P-B angle associated with the two Nd centers in the middle of the chain is 105.7°. Likewise, the bridging B-P-B angle are in good agreement with experiment ranging from 113.4 to 121.7°, compared to an average value of 120.6° in experiment. The oligomers based on a hypothetical **Nd-*i*Pr** polymeric structure also showed similar patterns in Nd-B distances and B-P-B angles (Tables S12-S15).

Table S8. Chelating Nd-B distances (Å) in the tetramer **Nd-Et-4** optimized with TPSS-D3/def2-SV(P) (def2-TZVP for Nd). Atom labels correspond to those in Figure S31.

**Bonds (side)**	**Distance**	**Bonds (side)**	**Distance**
Nd2-B124	2.81999	Nd1-B178	2.89388
Nd2-B153	2.78674	Nd1-B182	2.88325
Nd2-B183	2.84839	Nd4-B181	2.88771
Nd2-B179	2.79103	Nd4-B185	2.89211
Nd3-B213	2.80095		
Nd3-B150	2.78557		
Nd3-B180	2.74362		
Nd3-B184	2.79605		
**Avg.**	**2.797**	**Avg.**	**2.889**

Table S9. Bridging Nd-B distances (Å) in the tetramer **Nd-Et-4** complexes optimized with TPSS-D3/def2-SV(P) (def2-TZVP for Nd). Atom labels correspond to those in Figure S31.

**Bonds (longer)**	**Distance**	**Bonds (shorter)**	**Distance**
Nd2-B149	2.88286	Nd2-B119	2.61362
Nd4-B147	2.87026	Nd4-B121	2.65504
Nd4-B151	2.74031	Nd4-B123	2.67622
Nd1-B152	2.73107	Nd1-B122	2.72491
Nd1-B148	2.86667	Nd1-B118	2.63549
Nd3-B146	2.9065	Nd3-B120	2.63283
**Avg.**	**2.833**	**Avg.**	**2.656**

Table S10. Chelating B-P-B angles (°) in the tetramer **Nd-Et-4** complex optimized with TPSS-D3/def2-SV(P) (def2-TZVP for Nd). Atom labels correspond to those in Figure S31.

B-P-B Angle (side)		B-P-B Angle (middle)	
B124-P12-B153	110.95265	B181-P211-B185	105.93152
B183-P209-B179	111.24925	B182-P208-B178	105.47814
B150-P9-B213	112.91636		
B180-P210-B184	114.59284		
**Avg.**	**112.4**	**Avg.**	**105.7**

Table S11. Bridging B-P-B angles (°) in the hypothetical tetramer **Nd-^{i}Pr-4** optimized with TPSS-D3/def2-SV(P) (def2-TZVP for Nd). Atom labels correspond to those in Figure S32.

B-P-B Angle	
B119-P6-B147	116.11358
B149-P8-B121	121.73212
B118-P5-B146	122.4155
B148-P7-B120	115.91117
B151-P10-B122	113.41529
B123-P11-B152	114.3501
**Avg.**	**117.3**

Table S12. Chelating Nd-B distances (Å) in the hypothetical tetramer **Nd-^{i}Pr-4** optimized with TPSS-D3/def2-SV(P) (def2-TZVP for Nd). Atom labels correspond to those in Figure S32.

Bonds (side)	Distance	Bonds (middle)	Distance
Nd2-B125	2.82551	Nd4-127	2.83291
Nd2-B129	2.80976	Nd4-131	2.8557
Nd3-B126	2.7956	Nd1-124	2.87859
Nd3-B130	2.78301	Nd1-128	2.87822
**Avg.**	**2.803**	**Avg.**	**2.861**

Table S13. Bridging Nd-B distances (Å) in the hypothetical tetramer **Nd-^{i}Pr-4** optimized with TPSS-D3/def2-SV(P) (def2-TZVP for Nd). Atom labels correspond to those in Figure S32.

**Bonds (longer)**	**Distance**	**Bonds (shorter)**	**Distance**
Nd2-B101	2.81167	Nd2-B67	2.56934
Nd4-B99	2.82057	Nd4-B69	2.78951
Nd4-B102	2.83362	Nd4-B73	2.70137
Nd1-B104	2.85044	Nd1-B70	2.66462
Nd1-B100	2.84588	Nd1-B66	2.6311
Nd3-B98	2.79318	Nd3-B68	2.55221
**Avg.**	**2.826**	**Avg.**	**2.651**

Table S14. Chelating B-P-B angles (°) in the tetramer **Nd-^{i}Pr-4** optimized with TPSS-D3/def2-SV(P) (def2-TZVP for Nd). Atom labels correspond to those in Figure S32.

**B-P-B Angle (side)**		**B-P-B Angle (middle)**	
P125-B155-P129	109.32288	P127-B157-P131	107.14259
P126-B156-P130	111.52806	P124-B154-P128	105.9125
**Avg.**	**110.4**	**Avg.**	**106.5**

Table S15. Bridging B-P-B angles (°) in the tetramer **Nd-^{i}Pr-4** optimized with TPSS-D3/def2-SV(P) (Def2-TZVP for Nd). Atom labels correspond to those in Figure S32.

**B-P-B Angle**	
P67-B6-P99	118.14114
P69-B8-P101	116.61705
P73-B11-P104	119.4401
P70-B9-P102	121.32083
P68-B7-P100	120.32132
P66-B5-P98	118.37801
**Avg.**	**119.0**

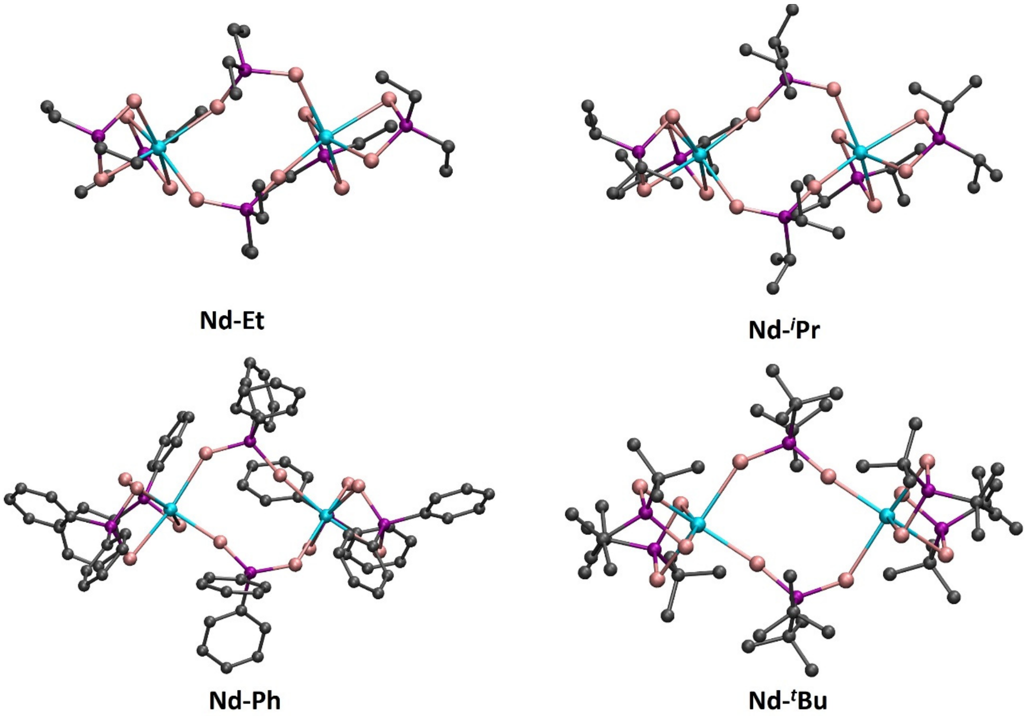

Figure S28. DFT optimized structure of **Nd-Et (model)**, **Nd-^{i}Pr**, **Nd-Ph**, and **Nd-^{t}Bu** complexes. Atoms are color coded as follows: Nd (cyan), B (pink), P (purple), carbon (gray). Hydrogen atoms are omitted for clarity.

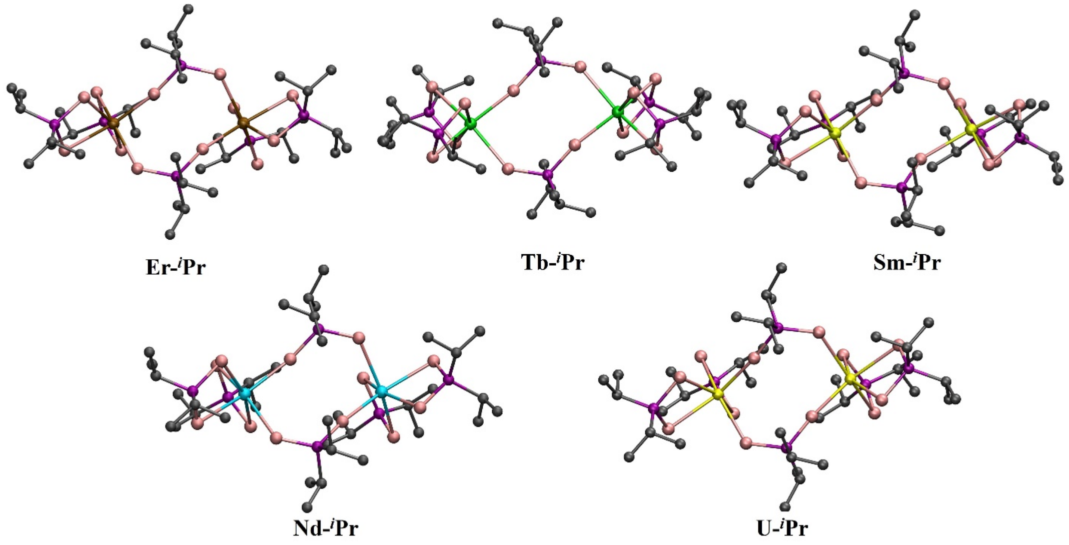

Figure S29. DFT optimized structures of **Er-^{i}Pr**, **Tb-^{i}Pr**, **Sm-^{i}Pr**, **Nd-^{i}Pr**, and **U-^{i}Pr** complexes. Atoms are color coded as follows: Er (brown), Tb (green), Sm (light yellow), Nd (cyan), U (yellow), B (pink), P (purple), carbon (gray). Hydrogen atoms are omitted for clarity.

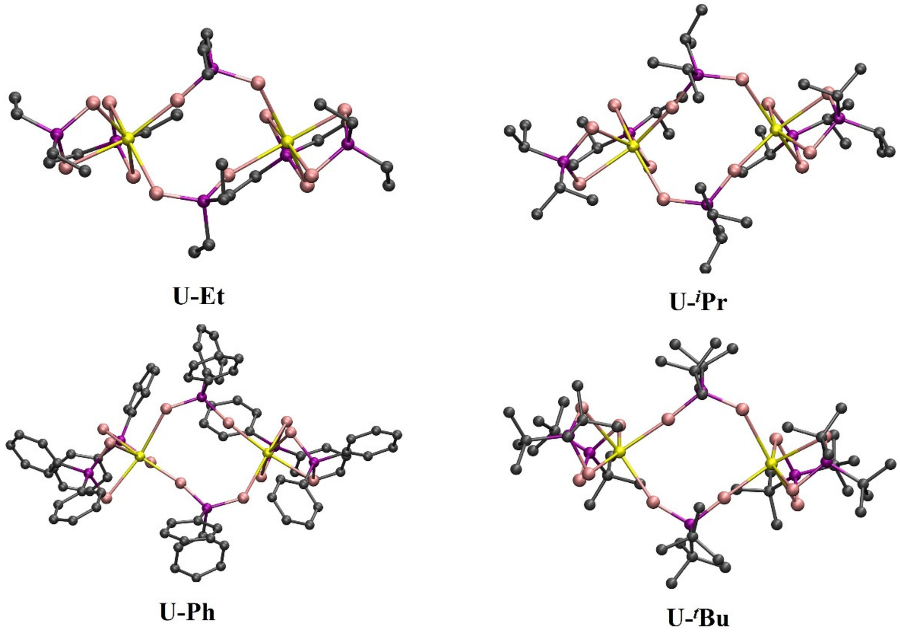

Figure S30. DFT optimized structure of **U-Et (model)**, **U-^{i}Pr**, **U-Ph**, and **U-^{t}Bu** complexes. Atoms are color coded as follows: U (yellow), B (pink), P (purple), carbon (gray). Hydrogen atoms are omitted for clarity.

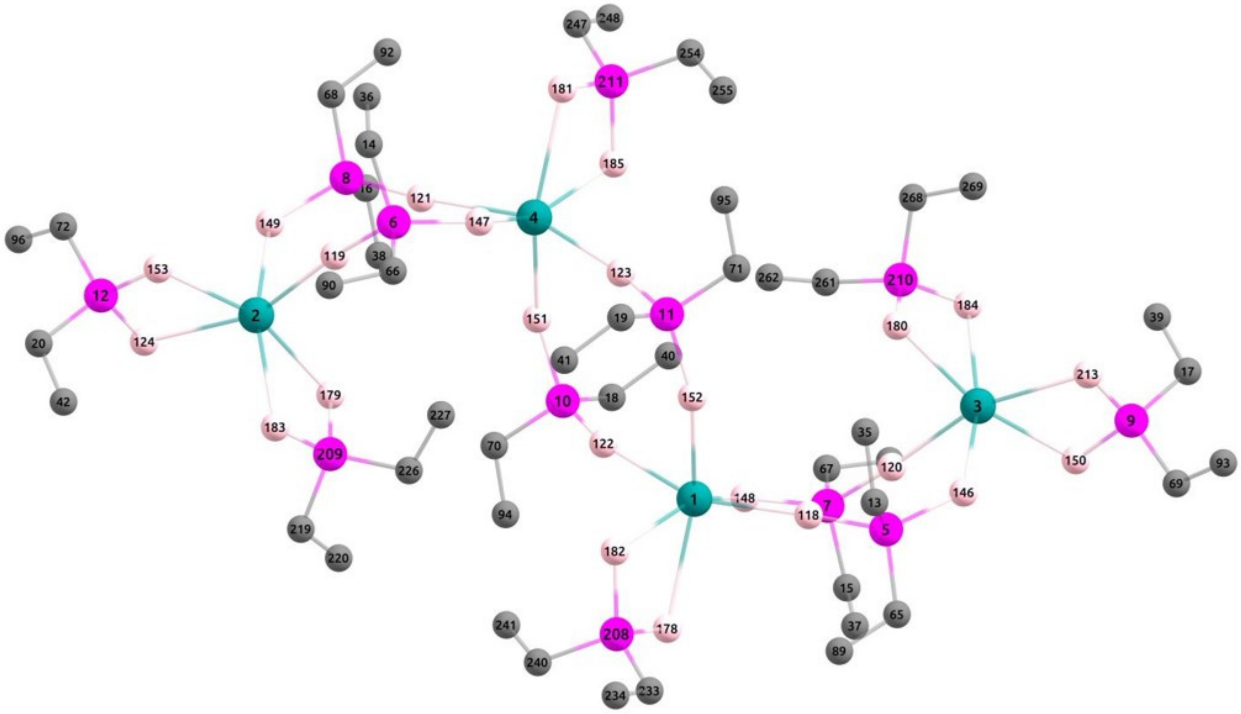

Figure S31. DFT optimized (TPSS-D3/def2-SV(P), def2-TZVP for Nd) **Nd-Et-4** complex with atom labels shown. Atoms are color coded as follows: Nd (cyan), B (pink), P (purple), C (gray). Hydrogen atoms are omitted for clarity.

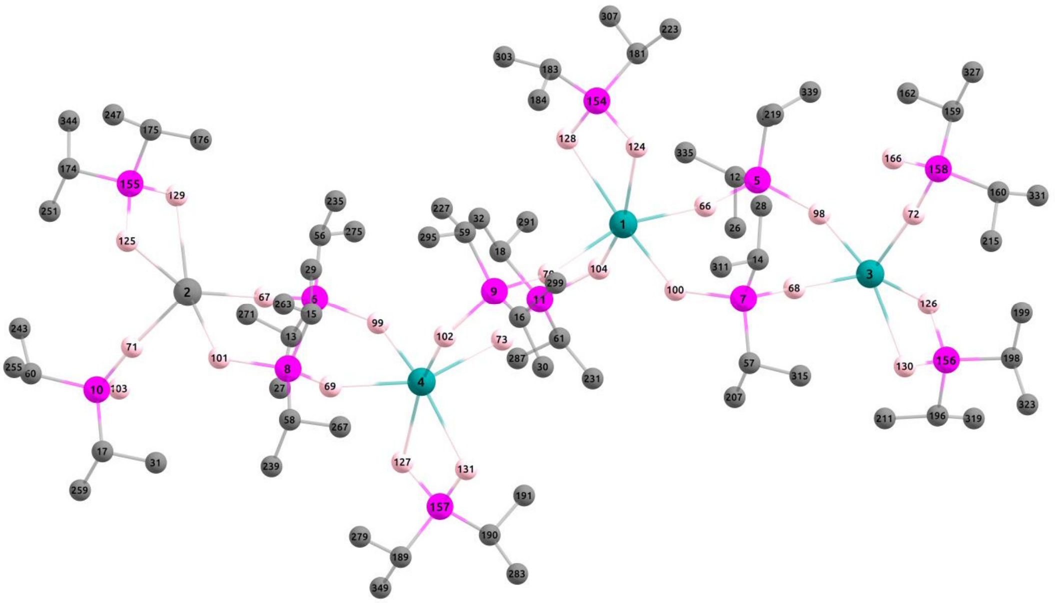

Figure S32. DFT optimized (TPSS-D3/def2-SV(P), def2-TZVP for Nd) **Nd-^{i}Pr-4** complex with atom labels shown. Atoms are color coded as follows: Nd (cyan), B (pink), P (purple), C (gray). Hydrogen atoms are omitted for clarity.
